# Surface Chemistry-Dependent Evolution of the Nanomaterial Corona on TiO_2_ Nanomaterials Following Uptake and Sub-Cellular Localization

**DOI:** 10.3390/nano10030401

**Published:** 2020-02-25

**Authors:** Abdullah O. Khan, Alessandro Di Maio, Emily J. Guggenheim, Andrew J. Chetwynd, Dan Pencross, Selina Tang, Marie-France A. Belinga-Desaunay, Steven G. Thomas, Joshua Z. Rappoport, Iseult Lynch

**Affiliations:** 1 Institute of Cardiovascular Science, College of Medical Dental Sciences, University of Birmingham, Birmingham B15 2TT, UK; a.khan.4@bham.ac.uk (A.O.K.); dcross501@gmail.com (D.P.); S.Thomas@bham.ac.uk (S.G.T.); 2 School of Biosciences, University of Birmingham, Birmingham B15 2TT, UK; A.DiMaio@bham.ac.uk; 3 School of Geography, Earth and Environmental Sciences, University of Birmingham, Birmingham B15 2TT, UK; E.J.Guggenheim@bham.ac.uk (E.J.G.); A.J.Chetwynd@bham.ac.uk (A.J.C.); M.A.Belinga@bham.ac.uk (M.-F.A.B.-D.); 4 Promethean Particles Ltd., 1-3 Genesis Park, Midland Way, Nottingham NG7 3EF, UK; selina.tang@proparticles.co.uk; 5 Boston College, Higgins 644A, 140 Commonwealth Ave, Chestnut Hill, MA 02467, USA

**Keywords:** nanosafety, protein corona, bionano-interface, cellular uptake, cellular localization, co-localisation, reflectance imaging

## Abstract

Nanomaterial (NM) surface chemistry has an established and significant effect on interactions at the nano-bio interface, with important toxicological consequences for manufactured NMs, as well as potent effects on the pharmacokinetics and efficacy of nano-therapies. In this work, the effects of different surface modifications (PVP, Dispex AA4040, and Pluronic F127) on the uptake, cellular distribution, and degradation of titanium dioxide NMs (TiO_2_ NMs, ~10 nm core size) are assessed and correlated with the localization of fluorescently-labeled serum proteins forming their coronas. Imaging approaches with an increasing spatial resolution, including automated high throughput live cell imaging, correlative confocal fluorescence and reflectance microscopy, and dSTORM super-resolution microscopy, are used to explore the cellular fate of these NMs and their associated serum proteins. Uncoated TiO_2_ NMs demonstrate a rapid loss of corona proteins, while surface coating results in the retention of the corona signal after internalization for at least 24 h (varying with coating composition). Imaging with two-color super-resolution dSTORM revealed that the apparent TiO_2_ NM single agglomerates observed in diffraction-limited confocal microscopy are actually adjacent smaller agglomerates, and provides novel insights into the spatial arrangement of the initial and exchanged coronas adsorbed at the NM surfaces.

## 1. Introduction

With the ever increasing prevalence of manufactured nanomaterials (NMs) and nanomedicines comes a pressing need for a comprehensive understanding of their interactions and behaviors in biological milieu [1,2,3]. The exposure of NMs to biological media results in adsorption events and the formation of a biomolecule corona, which is key to the subsequent fate of the NM [4,5,6,7,8,9,10,11]. Emerging data has shown a remarkable depth and complexity in the dynamic relationship between the composition of the biological environment and the physico-chemical properties of the NMs exposed therein [4,6,8,9,10,12]. Of these, surface chemistry is a vital component which impacts the corona composition and subsequent distribution, uptake, and clearance of NMs [4,6,8,9,10,12,13,14,15,16,17,18]. Indeed, a recent publication using the same particles as used here has shown that there are significant quantitative differences in the levels of fibrinogens, immunoglobulins, and several glycoproteins in the corona acquired from plasma [19]. These differences could correlate with different interactions with cell surface receptors, which have been observed previously, for example, with polysorbate-functionalized NMs preferentially adsorbing apolipoproteins aiding their passage across the blood–brain barrier [12]. Furthermore, Fleischer et al. demonstrated that cationic NMs acquire a protein corona that increases cellular binding, thought to be a result of a greater concentration of bovine serum albumen at the NM surface, which promotes interactions with scavenger receptors [20]. Finally, Mazzolini et al. demonstrated that transferrin in the NM corona may play a role in the clathrin-mediated endocytosis of NMs via transferrin receptors [21]. Therefore, it is likely that differences in the corona as a result of different polymer coatings may present different forms of receptor-mediated endocytosis.

Methods to determine corona evolution kinetics and the correlation of corona composition with NM uptake are vital for supporting NM safety-by-design [12,14,18]. Previous studies focusing on the NM corona have utilized mass spectrometry and fluorescent microscopy to identify components of the corona and develop models of their formation (and to a lesser extent loss) kinetics [22,23,24]. For example, the singular labeling of proteins of interest, such as the use of fluorescently-labeled bovine serum albumin (BSA), a significant component of the protein corona, has been applied. More recently, whole-serum fluorescent labeling approaches have been employed for studying the effect of the protein corona on nano-bio interactions, in order to assess the co-uptake of NMs and labeled corona, and as a post-hoc NM labeling technique for in vivo studies [9,23,24,25].

In this work, the impact of surface coatings with different charges and hydrophobicities on titanium dioxide (TiO_2_) NM uptake, localization, and protein corona loss or exchange dynamics are investigated. Previous studies of NM corona’s fate following cellular internalization have used model polystyrene particles, and the potential impact of coatings on the corona retention (or half-life) on NMs has thus not been considered [9,25]. TiO_2_ NMs are widely used, with various applications as pigments, food additives, components of medical and cosmetic products, catalytic agents, and drug delivery vectors, and in the treatment of water-borne pathogens [26,27,28,29,30,31,32,33,34,35,36]. TiO_2_ NMs are also known to make their way into the environment, often through routes as innocuous as the exposure of sun-screen and other cosmetic products, rich in TiO_2_ NMs, to bodies of water by swimmers, or similarly, into wastewater after bathing [29,36]. In the context of their widespread commercial, medicinal, and environmental presence, an improved understanding of the effects of the surface chemistry of coatings used to stabilize TiO_2_ NM on corona kinetics and subsequent cellular trafficking is critical to the safe design and effective application of these useful and versatile NMs.

Many inorganic NMs, including TiO_2_, are difficult to disperse in aqueous media, and thus polymeric coatings or other surfactant molecules are widely used to disperse and stabilize NMs in suspension, as well as to reduce the surface reactivity [6,37,38,39,40]. For example, the cytotoxicity of silver NMs has been reportedly modulated by coatings which alter the surface charge, binding to the cell surface, and aggregation and dissolution potential, which have been identified as major determining factors in cellular interactions and eliciting cytotoxicity [41]. Importantly, coatings, especially polyethylene glycol, have also been widely used to reduce protein binding to NMs, producing so-called “stealth particles” [38]. Three industrially relevant surface coatings are studied here: Polyvinylpyrrolidone (PVP), which has the potential to be positively charged in acidic pH conditions; Pluronic F127, a non-ionic polymer considered safe for cell culture; and Dispex AA4040, the ammonium salt of polyacrylic acid obtained via modification with alkylacrylates (see Supplementary Figure S1 for details and structures) [40].

**Figure 1:**
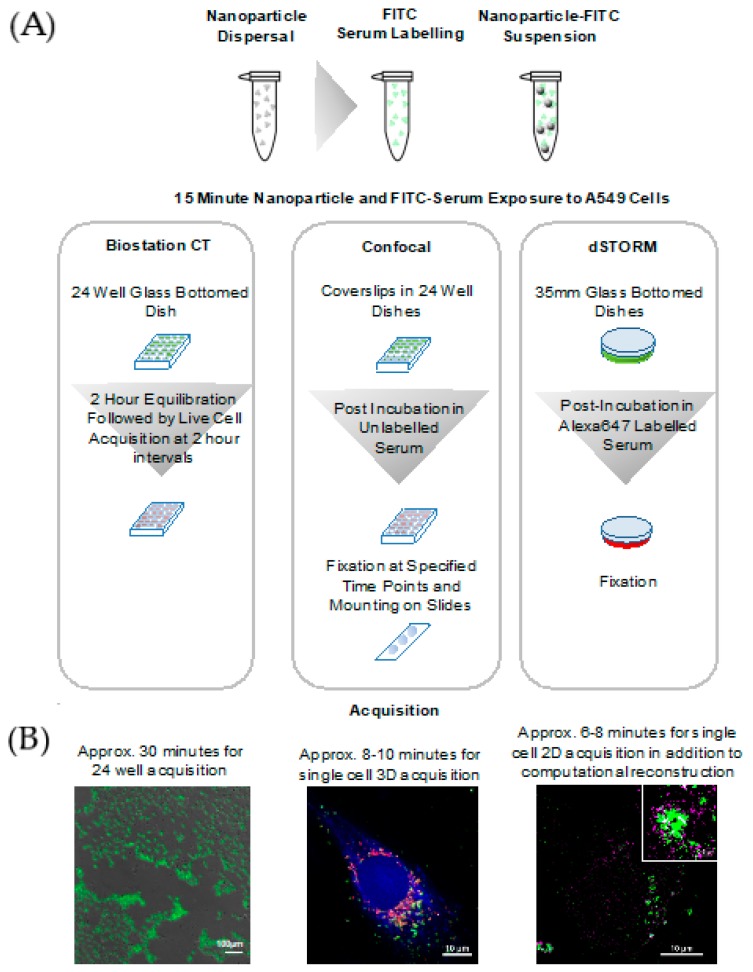
Imaging workflow applied to investigate the effect of the surface coating composition on the uptake, trafficking, and corona kinetics of titanium oxide (TiO_2_) nanomaterials (NMs). (**A**) Serum containing media is labeled with fluorescein isothiocyanate (FITC) prior to incubation with TiO_2_ NMs. (**B**) Adenocarcinomic human alveolar basal epithelial (A549) cells are then exposed to TiO_2_ NMs in FITC-serum for 15 min before subsequent washes and post incubation in either unlabeled serum (Biostation CT and confocal experiments) or Alexa-647-labeled serum (dSTORM). Images were acquired over relevant time points and analyzed for a quantitative comparison of the effects of different coating surface chemistries.

A range of imaging modalities were applied to study the effects of these surface coatings on the retention of fluorescently-labeled serum proteins, forming the protein corona, by TiO_2_ NMs internalized by adenocarcinomic alveolar human basal epithelial (A549) cells (Figure 1). An automated, high throughput live cell imaging system (Nikon Biostation CT) was employed as an epifluorescence microscopy screening tool. This live cell data was further supplemented by correlative fluorescence and reflectance confocal microscopy to provide an insight into the fate of unlabeled TiO_2_ NMs over time after initial cellular exposure. As many environmentally-, clinically-, and commercially-relevant NMs are electron dense, reflectance microscopy allows for the study of these particles in vitro, without the need for laborious labeling procedures, and perhaps more importantly, while retaining their native surface composition (and thus their acquired corona composition and stability) [42,43]. Finally, direct Stochastic Optical Reconstruction Microscopy (dSTORM) single-molecule super-resolution microscopy was applied to provide an examination of corona behaviors on an unprecedented nanoscopic scale of fluorescence image acquisition. The images presented here are the first evidence of an exchange of NM-surface bound proteins occurring in situ in cellular lysosomes, rather than simply the loss of proteins due to enzymatic degradation in the acidic lysosomal environment, as has been previously hypothesized [9,25].

## 2. Materials and Methods 

Cell culture: Adenocarcinomic human alveolar basal epithelial (A549) cells were obtained from the Health Protection Agency Culture Collection, UK. As an alveolar epithelial cell line, A549 cells are frequently used in nanotoxicology as a cell line representative of the respiratory tissues that would be primarily exposed to NMs [44]. A549 cells were grown in Dulbecco’s modified Eagle’s medium (DMEM, Thermo Fisher Scientific, Loughborough, UK) and supplemented with 10% fetal bovine serum (FBS, Gibco, UK), 1% penicillin streptomycin (Gibco, UK), and an additional 2 mM glutamine (Gibco, UK). A live cell serum containing medium was prepared for labeling experiments (described below), composed of 10% serum, 9.7 g Hanks Balanced Salt (without phenol red or sodium bicarbonate) (Sigma, Gillingham, UK), and 2.38 g HEPES (Thermo Fisher Scientific, Loughborough, UK), dissolved in 1 L of autoclaved, ultrapure MilliQ water. The media was then adjusted to pH 7.4 before sterile filtration through a 500 mL 0.1 µL pore vacuum filter system (Corning Life Sciences, Corning, NY, USA). 

Cells were grown and maintained at 37 °C in a 5% CO_2_ incubator. For Biostation CT and dSTORM experiments, cells were plated at 1.5 × 10^5^ on 24-well glass-bottomed dishes (MatTek Corporation, Ashland, MA, USA). For confocal experiments, cells were plated on coverslips (1.5 mm thickness, round 20 mm, VWR) in 24-well plates prior to fixation (described below) and mounting on slides (VWR).

Synthesis of TiO_2_ NMs: Titanium oxide (TiO_2_) NMs were synthesized by a hydrothermal technique using the continuous-flow reactor developed and patented by Lester et al., 2006 [45]. All NMs were manufactured by Promethean Particles Ltd. (Nottingham, UK) using this flow reactor. The process uses a metal salt solution pump (Gilson model 305/25.0 mL pump head) and an H_2_O pump (Gilson model 305/25.0 mL pump head) to deliver the precursors into the system. H_2_O was pumped through a pre-heating coil into the mixing point, where it was brought into contact with a concurrently flowing aqueous solution of titanium metal salt. The latter flow was pumped into the system at room temperature. The system pressure was maintained by a Dräger Tescom back-pressure regulator (Model 26-1700, UK). The temperature at the mixing point and the system pressure were maintained at supercritical conditions to ensure the formation of NMs. Under these conditions, the residence time was less than 9 s, assuming that the density of the reaction mixture was the same as pure water at the same temperature and pressure, and the total time from precursors being pumped in until sample collection was around 10 min. Using this method, TiO_2_ (anatase) NMs with a primary particle size of approximately 13 nm were produced. The mixture was cooled immediately after the mixing point using a series of heat exchangers. Functionalization was carried out in situ, after the first heat exchanger, whereby capping agents (polymers) were pumped in at a capping point downstream of the reactor. The capping agents were added at a concentration of 50 wt%, relative to the mass of the TiO_2_ NM product. The NMs were passed through the back-pressure regulator, returning the product stream to ambient pressure, and then collected as aqueous suspensions. The NMs were left to sediment over time (around 12 h on average), leaving a clear supernatant which was removed. The NMs were then washed, centrifuged, and re-suspended in fresh deionized water, resulting in aqueous stock suspensions at concentrations of ~2 wt% TiO_2_. These were stored at room temperature and shaken gently to reverse any settling prior to dispersion. 

NM dispersion: NM dispersions were prepared as described by the NanoMILE standard operating protocol (SOP), as shown in Supplementary Figure S2. TiO_2_ NMs were from Promethean Particles Ltd.: TiO_2_-un, TiO_2_-PVP, TiO_2_-F127, and TiO_2_-AA4040. All NMs had a core size of ~10 nm. 

Stock NM suspensions were diluted down to a working stock of 5 mg/mL in autoclaved, ultrapure MilliQ water, before aliquoting and sonication for 5 min, as described by the NanoMILE SOP (see Supplementary Figure S2 for details). Working stocks were diluted to 125 µg/mL for experiments. Fresh preparations were generated for each experiment.

Characterization of NM dispersions: Dynamic Light Scattering (DLS), zeta potential, and Inductively Coupled Plasma Mass Spectrometry (ICP-MS): NM stock suspensions were diluted as per the NanoMILE protocol for the dilution and dispersion of liquid suspension NMs in the relevant medium (ultrapure H_2_O, complete- serum containing medium (SCM), and artificial lysosomal fluid) to a concentration of 125 µg/mL [46]. Each NM suspension was then incubated at 37 °C in 5% CO_2_. Suspensions were characterized by DLS at specific time points (0, 2, 6, 18, and 24 h) post-dispersion and -incubation. At each time point, 800 µL was removed and measured by DLS. For DLS, suspensions were vortexed, and 800 µL of suspension was pipetted into a disposable DLS plastic cuvette. The diameter was measured using the NanoMILE Standard Operating Procedure (SOP) for TiO_2_ NMs. Measurements were taken a minimum of 10 times, with each sample consisting of three separate runs. Each condition was performed in triplicate. The results are presented as the mean of all measurements and the standard deviation (STD) of all measurements. Each NM also had zeta potential measurements taken in triplicate at 125 µg/mL at time 0 h in deionized water. The remainder of the sample was subsequently filtered through a Whatman filter with a pore size of 0.2 µm. HNO_3_ (ICP-grade) was then added to each sample to a final concentration of 2.5%, prior to ICP-MS measurement. 

ICP-MS measurements were run on a Perkin Elmer Nexion 300 in order to assess whether any dissolution of the NMs had occurred. Appropriate calibration curves were created for TiO_2_ using ICP-grade TiO_2_ standard in the appropriate matrix (water, artificial lysosomal fluid (ALF), or SCM, in 2.5% HNO_3_). Calibrations were considered acceptable when the R^2^ correlation coefficient was greater than 0.999. Quality Checks (QCs) were performed throughout and analyzed every 15 samples. All ICP-MS samples were co-infused with Scandium as an internal standard and Ti responses were automatically adjusted to account for variation in the internal standard response.

Serum labeling and treatment: Serum labeling was performed using a ProtOn labeling kit (Vector Labs, Burlingame, CA, USA), with the staining solution prepared as per the manufacturer’s instructions. In brief, 750 µg desiccated fluorescein isothiocyanate (FITC) was suspended in 15 µL dimethylformamide (DMF) and stored at −20 °C until use. FBS was stained with ProtOn FITC stock at a concentration of 37.5 µg/mL and incubated for 2 h at room temperature, with regular mixing. To terminate the reaction, an equal volume (75 µL) of 1M ethanolamine was added to labeled serum and incubated at room temperature for 5 min. 

Diluted NMs (125 µg/mL) were allowed to equilibrate in FITC-serum for 20 min at 37 °C before exposure to cells for 15 min in a 37 °C incubator, after which FITC-serum was aspirated and 2 phosphate buffered saline (PBS) washes were performed. A 15-min post-incubation in either unlabeled serum or Alexa-647-labeled serum (dual staining experiments) was then performed. Cells were imaged (Time 0) and monitored over the subsequent 18 h to assess the trafficking of NMs and fate of the NM-protein coronas.

Alexa-647 labeling of serum was performed using Alexa-647NHS Ester (Succinimidyl Ester) (Thermo Fisher) at an equal concentration (37.5 µg/mL) in a 60 min incubation period at room temperature with regular mixing, as per the manufacturer’s instructions.

Cell staining and fixation: Cells for preliminary experiments were stained with 1 mM Lysotracker Red-DND 99 for 15 min prior to washing in PBS (Lonza, Basel, Switzerland) and fixation in 4% paraformaldehyde. Ready Blue Hoechst Stain (Thermo Fisher Scientific, Loughborough, UK) was applied post-fixation to stain nuclei, and cover slips were mounted in Vectashield Mounting Medium (Vector Labs, CA, USA). 

For Biostation CT and confocal experiments, cells were stained with CellTrace Violet (Thermo Fisher Scientific, Loughborough, UK) 4 h prior to experiments at 5 µM in PBS for 20 min prior to washing and post-incubation in serum-containing media. In confocal experiments, cells were also stained with Lysotracker Red as previously described, 15 min prior to the end of a time point and subsequent fixation. 

Biostation CT imaging: Automated live cell imaging was performed with a Nikon Biostation CT demo unit maintained at 37 °C and 5% CO_2_. Images were acquired with a 10X/0.30 (Plan Fluor Series) objective at 2 h intervals using phase contrast optics and a CFP cube (CellTrace Violet) and GFP/Alexa 488 cube (FITC).

Confocal imaging: Multi-channel confocal images were taken at the Birmingham Light Advanced Microscopy (BALM) facility at the University of Birmingham, UK. To allow a proper comparison of different time points, the same optical configuration (digital zoom, photomultiplier tube, laser power, Galvano scanner, and pinhole) was employed in all acquisitions. Samples were imaged by using the 402 (diode laser), 488 (Ar/ArKr laser), 561, and 636 nm (He/Ne laser) laser lines on a Nikon A1R laser scanning confocal system mounted on a Nikon Ti inverted microscope with a 100X/1.49 oil objective (Nikon, Tokyo, Japan). The pinhole size was set to 1 AU, the digital zoom was set to 2.18, and an averaging (2 scan accumulations) mode was used to improve the signal to noiuse ratio (S/N). NMs were imaged using a reflectance configuration consisting of a 488 nm laser, a BS20/80 dichroic mirror, and a 560/40 filter cube (centered at 560 nm with a 40 nm bandpass). For all channels, a series of representative optical sections were collected. Post-imaging analyses were performed using Fiji (ImageJ2) and Nikon Elements 4.2 (Nikon, Japan) software. Imaging conditions were maintained across all experiments, with the brightness only being adjusted for purposes of presentation.

dSTORM imaging: Direct Stochastic Optical Reconstruction microscopy (dSTORM) was performed using a Nikon N-STORM system on a Ti-E stand with Perfect Focus, an Andor iXon Ultra DU-897U EMCCD camera, and an Agilent MLC400 high-power laser bed. Images were acquired through a 100X 1.49 NA TIRF Objective on the system, with 20,000 frames acquired through NIS Elements 4.2 (Nikon, Japan). Fluorophore blinking required for stochastic reconstruction was induced by imaging in a PBS buffer comprised of 50 µg/mL glucose oxidase, 1 µg/mL catalase, and 100 mM mercaptoethylamine-HCL. Images were reconstructed through the Nikon STORM analysis module (v 3.2, Nikon, Japan), with drift correction, Gaussian rendering, and a density filter (15 mol/200 nm radius).

Image and data analysis: Image analysis was performed on NIS Elements High Content Analysis (v 4.3.002, Nikon, Japan) using the General Analysis module with the JOBS explorer (segmentation workflow as per Supplementary Figure S6). Statistical analysis and graphical presentation were performed in GraphPad PRISM (Version 6, GraphPad Software, San Diego, CA, USA). 

Individual channels of STORM images were clustered using the DBSCAN algorithm, with the minimum number of points constituting a cluster varying from 3 to 100 (min. points 1 and 2 were rejected due to being less than the dimensionality of the data + 1). A total of 5–6 images across three replicates for each time point and condition were analyzed. Each partitioning was validated using the inter-cluster variance to determine the optimal cluster labels. The alphaShape Matlab function was employed to construct a concave hull around each set of cluster points; from this, the volume of clusters was extracted and used to calculate the density of points within the cluster. Clusters were visualized using the gscatter Matlab function to produce a scatter plot of points color-coded according to cluster membership, with the alpha shapes plotted over these points to show the cluster structure.

Statistical analysis and graphing were performed using GraphPad PRISM (Version 6, GraphPad Software, USA).

TEM imaging: Samples were prepared for Transmission Electron Microscopy (TEM) to determine the average core size of NMs. A drop of diluted NM suspension was partially dried on a copper mesh 400 holey carbon Film (Agar Scientific, Stansted, UK), before washing with UHP water, and finally re-dried. Images were acquired by means of a JEOL 1200EX (with an accelerating voltage of 80 kV) and recorded with Gatan Digital Micrograph Software (GMS3.x, Gatan, Inc., Pleasanton, CA, USA). The analysis of core size was performed by Image J (Version 2, NIH, Bethesda, MD, USA).

## 3. Results and Discussion

### 3.1. Fluorescein Isothiocyanate (FITC)-Serum Labeling and High Throughput Live Cell Screening of Corona Interactions in TiO_2_ NM-Containing Cells with Different TiO_2_ Surface Modifications

Initial experiments to test the labeling of FITC serum were performed using uncoated (TiO_2_-un) and Dispex-AA4040-coated (TiO_2_-AA4040) TiO_2_ NMs, as representatives of bare and surface-modified NMs, respectively. Both NMs had an average core size of 12–14 nm (Supplementary Figure S3). Dynamic Light Scattering (DLS) and Inductively Coupled Plasma Mass Spectrometry (ICP-MS) measurements showed no significant changes in particle size over time in the serum-containing medium (Supplementary Figure S4), although changes in the agglomerate size were observed in artificial lysosomal fluid (ALF), suggesting that the changes observed in the live cell imaging were likely the result of uptake and trafficking-related changes in agglomerate size, followed by changes arising in the lysosomes. Complete live cell imaging media with 10% fetal bovine serum (FBS) was labeled as described in the materials and methods, before pre-incubation with NMs and a subsequent 15 min exposure to A549 cells. A 15 min post-incubation procedure in unlabeled serum-containing media was then performed. 

Effective labeling of serum proteins was demonstrated (Supplementary Figure S5), and in NM-treated samples, the FITC signal co-localized well with the reflectance signal (* *p* = 0.0202), indicating the adsorption of fluorescent proteins onto the NM surface to form a protein corona after a 15 min incubation period in FITC-serum. The FITC signal in control cells was nearly undetectable (*p* < 0.0001) when compared to NM-treated samples and, in the majority of cells, could be removed through thresholding, indicating that in treated cells, FITC was transported into cells as a result of labeled protein binding of the NMs (Supplementary Figure S5). Some FITC spots of a higher intensity were observed in a minority of control cells; however, these did not correlate with the reflectance signal, suggesting that further analysis could effectively and robustly discriminate between the background and corona by restricting measurement to FITC directly associated with reflectance spots, as indicated in Supplementary Figures S6 and S7. Similarly, NMs demonstrated significant agglomeration at the cell surface, suggesting that further experiments should employ cell staining to allow for an effective segmentation of the interior of the cell to permit the assessment of internalized NMs only (Supplementary Figure S7). 

A live cell screening approach utilizing the Biostation CT was then performed (see Figure 1). The initial FITC-serum intensities were considered as indicative of initial corona binding to uncoated TiO_2_ NMs (TiO_2_-un) and surface-coated variants TiO_2_-PVP, TiO_2_-F127, and TiO_2_-AA4040. Complete medium labeled with FITC which was not exposed to NMs was used as a serum control. Changes in the FITC signal over time were then assumed to indicate the evolution of the NM-protein corona, likely as a result of a loss of proteins via enzymatic degradation in the lysosomes, as has been shown previously for polystyrene-bound proteins [9].

**Figure 2:**
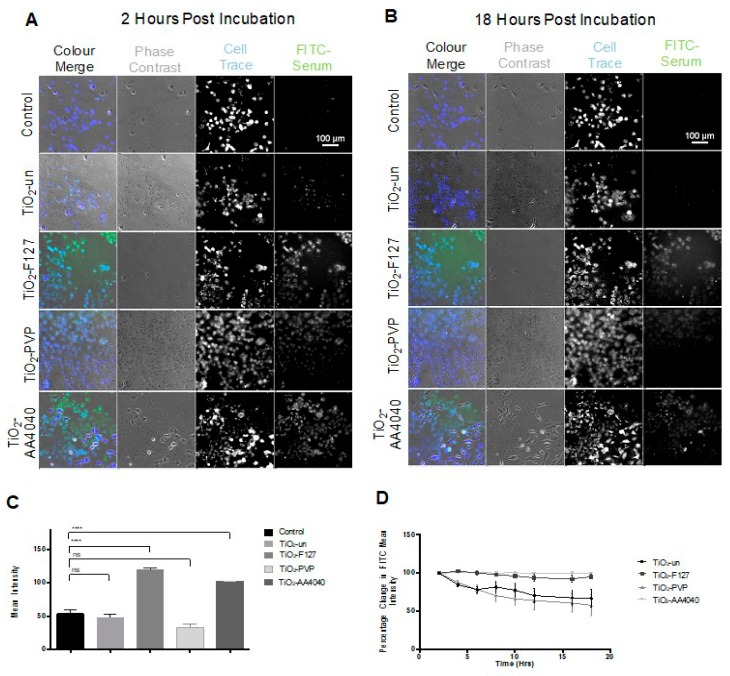
Biostation CT-acquired images and data showing the FITC-labeled serum intensity in A549 cells observed between 2 and 18 h post-exposure to FITC-labeled serum-containing medium in the absence of NMs (control) or with uncoated (TiO_2_-un), F127 (TiO_2_-F127), PVP (TiO_2_-PVP), or AA4040 Dispex-coated (TiO_2_-AA4040) TiO_2_ NMs. (**A**) At 2 h post-incubation, a significant green signal is retained versus controls treated with FITC-serum in the absence of NMs; (**B**) at 18 h post-incubation, there is a significant loss of the FITC signal in TiO_2_-un- and TiO_2_-PVP-treated samples, while TiO_2_-F127 and TiO_2_-Dispex treatments exhibit a much smaller change. (**C**) The total raw mean FITC intensity data averaged over time showed statistically significant differences in intensity between the control, TiO_2_-F127, and TiO_2_-AA4040 NMs when treatment groups were compared to control with an unpaired t-test (*p* < 0.001). Significance was not observed when controls were compared to cells treated with TiO_2_-un and TiO_2_-PVP NMs. (**D**) Normalizing mean intensity data to a percentage change in signal between subsequent time points shows no significant change in the FITC-serum intensity in TiO_2_-AA4040- and TiO_2_-F127-treated cells, suggesting the retention of an FITC-labeled serum corona. Comparatively, TiO_2_-un- and TiO_2_-PVP-coated NMs show a progressive loss of FITC signal over the time observed. Log transformation of the percentage change allows for analysis of the data set with a repeated measures ANOVA test indicating significance (*p* = 0.0250) in variance across NMs, and significance (*p* = 0.018) in variance across time (two-way ANOVA, SEM, *n* = 3).

As shown in Figure 2A,B, there is a notable difference in the surface fluorescence intensity when comparing uncoated and surface-modified TiO_2_ NMs at 2 and 18 h post-treatment, indicative of different corona fates, depending on the NM coating. There is no significant difference in the total mean FITC intensity when comparing TiO_2_-un and TiO_2_-PVP to FITC serum controls (Figure 2C), suggesting a rapid loss of FITC-labeled proteins from the NM surfaces; however, a significantly higher FITC signal is observed in TiO_2_-F127 and TiO_2_-AA4040 NM-treated A549 cells compared to the controls (*p* < 0.001), indicating retention of the FITC-labeled proteins in these NM coronas over the duration of the experiment. Interestingly, measurements of the FITC-serum intensity at several time points over the 18 h period show markedly different trends between the various coated TiO_2_ NMs (Figure 2D), consistent with Figure 2C. A two-way ANOVA showed significance in variance across NMs (*p* = 0.0250) and time (*p* = 0.018). While the FITC-serum intensity decreases by 43.18% (SE ± 3.84) and 61.01% (SE ± 1.246) in TiO_2_-un and TiO_2_-PVP, respectively, the signal is retained in TiO_2_-F127- and TiO2-AA4040-treated cells (decreasing by only 4.75% SE ± 5.128 and 0.56% SE ± 0.2207, respectively). The significantly higher FITC-serum signal retained by TiO_2_-F127 and TiO_2_-AA4040 NMs suggests the formation of large NM agglomerates which retain their initial corona proteins, as indicated in Supplementary Figure S8 for TiO_2_-AA4040. The decreased signal observed in uncoated and PVP-modified TiO_2_ NMs potentially suggests a rapid loss of the initial corona, indicating a difference in corona retention over time as a function of the NM coating composition. 

The screening data set presented a number of significant analytical challenges. Firstly, within a live cell format, there is a notable loss of fluorescence from cells over time, presenting difficulties in terms of accurately distinguishing the NM-associated FITC-serum signal from the background. The washing and mounting steps employed in the preliminary work would have significantly reduced the background, while live cell samples would be subject to the normal trafficking of FITC-labeled serum protein which remains un-associated with the incubated NMs. This issue is further compounded by the low resolution and lack of optical sectioning (which is offered by confocal microscopy) in this high throughput epifluorescence system. As such, out-of-focus light will play a role in increasing the background. To overcome issues with out-of-focus light, a similar set of data was generated using confocal imaging, for comparison. 

### 3.2. Confocal Investigation of the Uptake and Trafficking of TiO_2_ NMs with Different Surface Coatings

Confocal experiments were designed to further interrogate the FITC-serum corona data generated from the Biostation CT screening. Confocal imaging offers a higher resolution, optical sectioning, and the capacity for correlative fluorescence and reflectance studies, allowing both the labeled proteins and the NMs to be tracked and localized in parallel. With appropriate analytical approaches, insights into the bioavailability of large TiO_2_ NM agglomerates and their subsequent internalization and co-localization with fluorescently-labeled lysosomes can be determined and studied alongside any changes in observed corona kinetics. 

To this end, cells were stained with Cell Trace Violet prior to treatment with FITC-serum-incubated TiO_2_ NMs, as previously described. Fifteen minutes prior to fixation at 0, 2, 6, 18, and 24 h post-treatment cells were further stained with Lysotracker Red for co-localization studies. A fixed cell approach was used here to reduce the background fluorescence, as observed in live cell imaging, and to remove the possibility of FITC photo-bleaching through repeated acquisitions.

**Figure 3:**
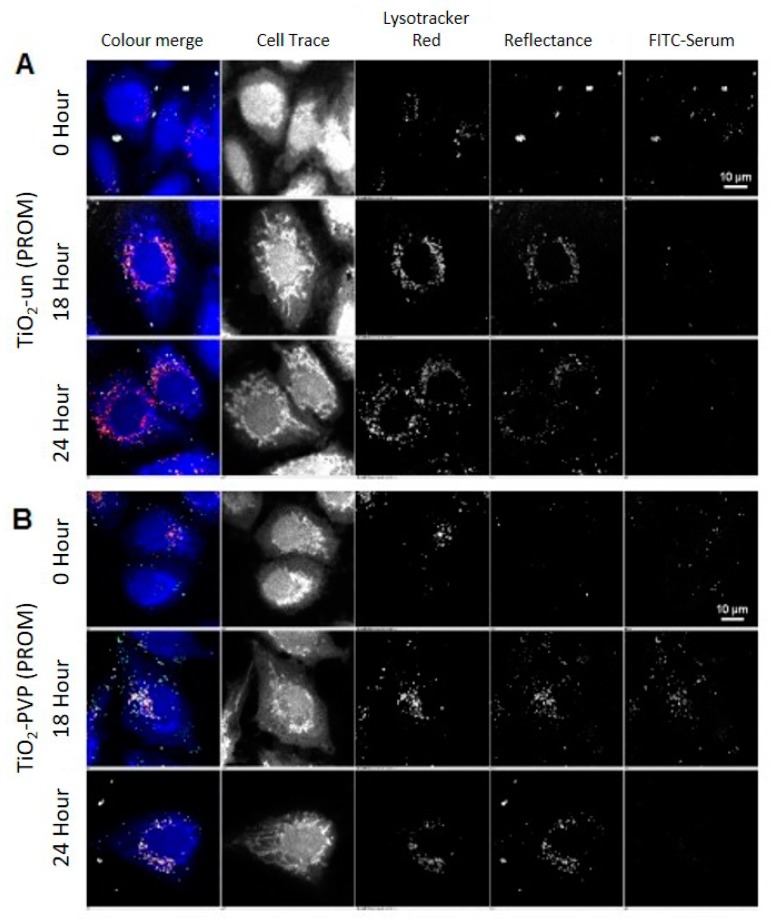
Confocal acquisition of A549 cells treated with uncoated (TiO_2_-un) and PVP-coated (TiO_2_-PVP) TiO_2_ NMs compared to the control (untreated cells) in FITC-labeled serum, followed by post-acquisition in unlabeled serum and fixation at 15 min (0 h), 2, 6, 18, and 24 h post-exposure. (**A**) Images show the presence of TiO_2_-un as large, bright agglomerates associated with the surface of the cell, which are subsequently internalized and trafficked to lysosomes marked by lysotracker red. Past the 0 h time point, TiO_2_-un rapidly loses the FITC signal, suggesting that in the absence of a surface coating, the protein corona is not stably bound. (**B**) TiO_2_-PVP, on the other hand, demonstrates an increase of the FITC signal up to the 18 h time point, after which there is a significant loss of signal. As NMs co-localize well with lysotracker at 18 and 24 h, this is likely due to degradation of the protein corona within the acidic lysosomal compartment.

**Figure 4:**
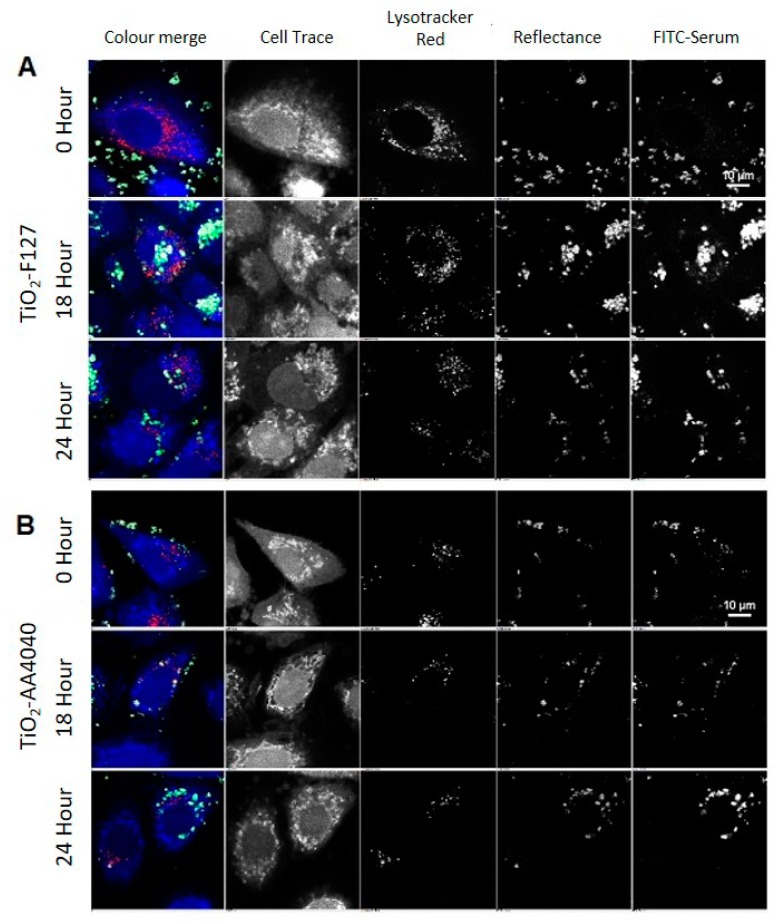
Confocal acquisition of A549 cells treated with (**A**) Pluronic-F127 (TiO_2_-F127) and (**B**) Dispex-AA4040 (TiO_2_-AA4040)-coated TiO_2_ NMs and compared to the control (untreated cells) in FITC-labeled serum, followed by post-acquisition in unlabeled serum and fixation at 15 min (0 h), 2, 6, 18, and 24 h post-exposure. Compared to TiO_2_-un- and TiO_2_-PVP-coated NMs (Figure 3), these show significantly larger agglomerates which accumulate on the cell surface, and have been taken up into the cells by the 18 h time point, beyond which they appear to be retained.

Images were acquired as representative Z-stacks and processed with the High Throughput Analysis Module of NIS Elements, as detailed in Figure S7. Reflectance imaging shows that TiO_2_ NMs are initially exposed to cells as large agglomerates (as per the TEM and DLS data shown in Supplementary Figures S3 and S4, respectively), which are subsequently internalized over the 24-h period observed (Figure 3 and Figure 4). Interestingly, the surface coating has a distinct effect on retention of the FITC-serum initial corona, and the apparent size of the internalized TiO_2_ NM agglomerates, consistent with the DLS data in ALF, which shows a much smaller agglomerate size for the uncoated NMs compared to the coated ones (Supplementary Figure S4). 

TiO_2_-un and TiO_2_-PVP are internalized as small discrete agglomerates which are trafficked to lysosomes in the time observed (Figure 3A,B). A key difference here is that TiO_2_-un appears to rapidly lose FITC-serum, while TiO_2_-PVP retains the labeled corona until 18 h. TiO_2_-F127 and TiO_2_-AA4040 demonstrate different agglomeration and internalization kinetics, and appear as larger structures which retain an FITC-serum corona at 18 and 24 h (Figure 4A,B). Interestingly, TiO_2_-AA4040 appears to be internalized more significantly at 24 h. 

**Figure 5:**
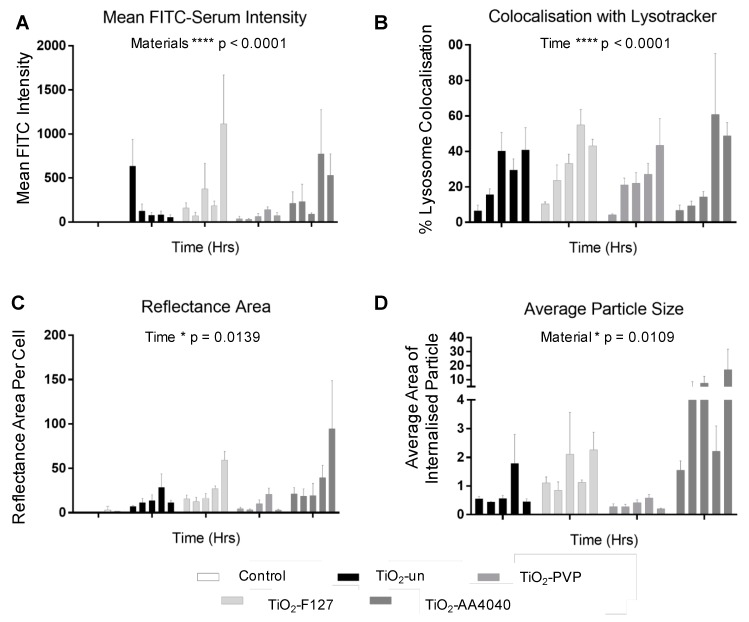
(**A**) Quantification of confocal data at 0, 2, 6, and 24 h post-exposure shows a change in the FITC-serum signal over time. While TiO_2_-un NMs rapidly lose the FITC-serum signal, surface-modified particles retain this, with a steady increase over time. TiO_2_-PVP demonstrates a slight reduction in signal between 18 and 24 h, potentially indicative of lysosomal degradation. (**B**) All four NMs demonstrate a steady increase in lysosomal co-localization, with significance demonstrated over time (**** *p* < 0.0001), but not between materials. (**C**) An increase in the reflectance area over time, indicative of NM uptake, is observed (* *p* = 0.0139), with no significant difference in NMs observed, consistent with the equivalent exposure of each NM type to the cells in question. (**D**) Finally, a difference in NM agglomerate size (in microns) is noted between materials (* *p* = 0.0109) consistent with images which show TiO_2_-PVP internalized as small discrete agglomerates, compared to the larger agglomerates of TiO_2_-F127 and TiO_2_-AA4040 observed (two-way ANOVA, SEM, *n* = 3).

Quantification of this confocal data set reveals a significant difference (*** *p* < 0.0001) across materials. TiO_2_-un rapidly loses its associated FITC-serum corona (Figure 5A), despite a progressive increase in the reflectance area and lysosomal co-localization, with both indicating continued internalization and trafficking (Figure 5B,C). Conversely, surface-modified NMs retain the FITC-serum corona over the observed time period, with an increase in the FITC-serum intensity, lysosomal co-localization, and reflectance area over time (Figure 5A–C). Note that after the initial 15 min of exposure to the NM-containing medium, there was no further exposure to NMs and so evolution of the NM concentration observed through reflectance was likely due to the reorganization of NMs within the cells via trafficking rather than additional uptake.

All NMs observed exhibited a significant increase in co-localization with lysosomes over time (*p* < 0.0001). Despite this, only TiO_2_-PVP demonstrated a reduction in FITC-serum intensity, reflectance area, and average particle size (Figure 5B–D) consistent with lysosomal degradation. This data suggests that TiO_2_-PVP is more readily trafficked and degraded than TiO_2_-un, TiO_2_-F127, and TiO_2_-AA4040. Finally, a significant difference in the average NM agglomerate size was observed across materials (* *p* = 0.0109), with TiO_2_-PVP NM agglomerates being notably smaller throughout the experiment compared to the other surface-modified NMs observed. TiO_2_-AA4040 NM agglomerates were observed to be the largest. 

These results expand on the live cell Biostation data, and the addition of NM reflectance and lysosomal data provides further insight into the fate of internalized TiO_2_ NMs and NM-agglomerates. Surface modification appears to improve the retention of the FITC-serum initial corona, and in the case of TiO_2_-F127 and TiO_2_-AA4040 NM, impacts the agglomeration behavior and trafficking in A549 cells.

### 3.3. Dual-Stained Corona and dSTORM Super-Resolution Imaging

Surface modification with different coatings appears to alter the ability of TiO_2_ NMs to retain their initial FITC-serum signal following cellular internalization. A key question is whether the loss of the FITC-serum signal observed was due to an exchange of labeled proteins at the NM surface with unlabeled proteins present in the endosomes and lysosomes, or the product of the acidic degradation of proteins (and subsequent loss of FITC) within lysosomes as NMs were internalized and trafficked.

To shed light on this question, a dual-staining experiment was performed whereby samples were initially exposed to FITC-labeled serum, as previously described, to allow NM uptake, after which the cells were post-incubated for 15 min in medium-containing serum proteins labeled with an alternative fluorophore (Alexa-647). This combination of the cellular uptake of NMs with their initial coronas containing FITC-serum proteins, followed by incubation of the NM-containing cells in Alexa-647-labeled serum-containing medium, allows for an assessment of both initial corona loss and exchange with external serum proteins that have been independently trafficked into the cells.

**Figure 6:**
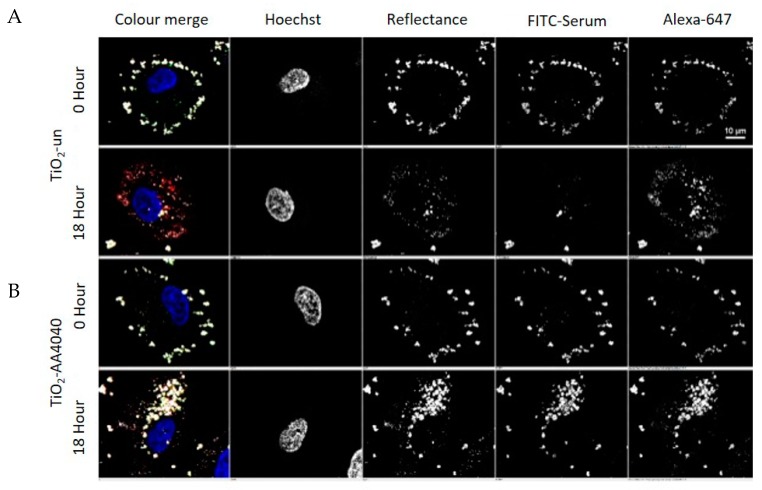
A549 cells incubated with TiO_2_ NMs in FITC-labeled serum, followed by a post-incubation period in Alexa-647-labeled serum for 15 min (0 h) and 18 h prior to fixation and confocal imaging. (**A**) Uncoated TiO_2_ NMs (TiO_2_-un) demonstrate a loss of the FITC signal by 18 h, suggesting a loss of the initial corona. Alexa-647-labeled serum remains bound to TiO_2_-un at 18 h, suggesting that the loss of the initial corona signal is likely to be a consequence of protein exchange. (**B**) AA4040 Dispex-coated TiO_2_ NMs (TiO_2_-AA4040) demonstrate markedly different spot sizes after internalization at 18 h and retain their initial corona, suggesting that surface modification can play a significant role in the corona exchange kinetics and uptake of TiO_2_ NMs.

A preliminary trial of this approach demonstrated a remarkable difference between TiO_2_-un and TiO_2_-AA4040 NMs at 0 and 18 h (Figure 6). Firstly, both FITC- and Alexa-647-labeled serum adsorb onto TiO_2_-NMs at 0 and 18 h. Interestingly, TiO_2_-un demonstrates a loss of the FITC-serum signal at 18 h, as indicated previously; however, there appears to be retention of the Alexa-647 signal, which was acquired post-uptake. TiO_2_-AA4040 NMs once again evidence the internalization of large intracellular agglomerates which retain both the FITC and Alexa-647 signal at 18 h post-treatment.

From the confocal evidence that such an approach may yield further insight into corona formation, retention, exchange, and/or degradation, direct Stochastic Optical Reconstruction Microscopy (dSTORM) was performed on TiO_2_-un, TiO_2_-PVP, and TiO_2_-AA4040 NMs, each of which exhibit a particular, distinct pattern of NM internalization and corona retention. TiO_2_-F127 did not show statistically significant differences to TiO_2_-AA4040, and as such, the latter, which exhibits the highest FITC-serum signal at 18 h, was taken forward for super-resolution imaging. Figure S9 shows the dSTORM controls for the two labeled sera (FITC and Alexa-647), indicating the clustering of labels in the presence of NMs compared to the diffuse background in the controls with no NMs.

Sub-diffraction limited imaging can reveal the relationship between fluorescently-labeled initial and exchanged coronas on a previously unprecedented nanoscopic scale. Indeed, Supplementary Figure S10 shows that NM agglomerates detected by Biostation and confocal microscopy could be resolved into smaller, closely associated agglomerates, as well as providing information about the corona dimensions and density. Figure 6 shows that TiO_2_-un NMs form the large extracellular agglomerates previously reported by confocal imaging (FITC-corona in green, Alexa-647 in magenta). 

**Figure 7:**
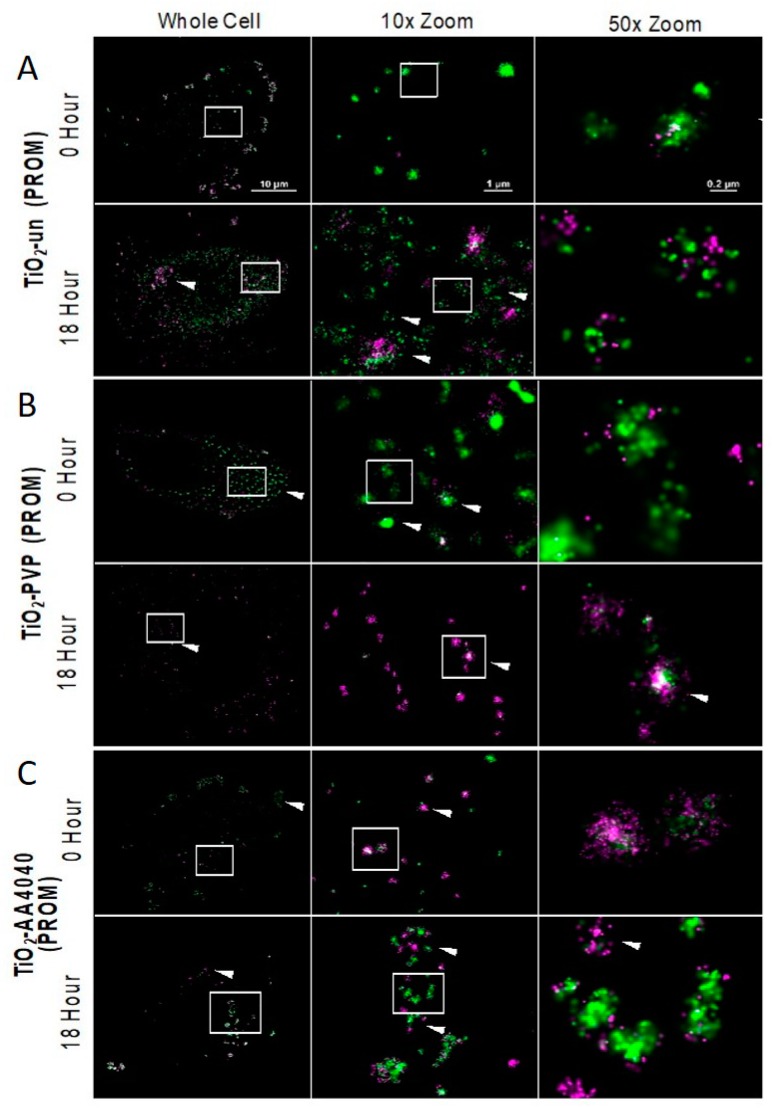
STORM acquisitions of A549 cells treated with TiO_2_ NMs. (**A**) Uncoated TiO_2_ at 0 and 18 h, (**B**) TiO_2_-PVP at 0 and 18 h and (**C**) TiO_2_-dispex at 0 and 18 h. At 0 h, uncoated (TiO_2_-un), PVP-coated (TiO_2_-PVP), and Dispex-AA4040 (TiO_2_-AA4040)-coated NMs demonstrate different behaviors. TiO_2_-PVP NMs are internalized as discrete, spherical spots which strongly associate with FITC-labeled protein (initial corona). In comparison, uncoated and dispex-AA4040-coated NMs form large agglomerates which associate with both FITC protein and Alexa-647 protein, forming an initial (green) and exchanged (magenta) corona. At 18 h post incubation, TiO_2_-PVP NMs have adsorbed a secondary layer of protein and formed larger agglomerates. Conversely, TiO_2_-un NMs appear to have lost the FITC-serum signal, and are instead associated with Alexa-647-labeled proteins. Interestingly TiO_2_-AA4040 NMs are internalized as large agglomerates which retain their initial corona (FITC) signal at the later time point observed. Reconstructions were performed using the NIS Element STORM module, with a density filter restricting molecules to clusters of 15 molecules within 200 nm.

By 18 h, these large agglomerates have been internalized as smaller, more discrete clusters, with FITC-serum molecules more loosely associated within the cluster. dSTORM imaging reveals that some of these clusters were comprised of Alexa-647-labeled serum proteins, suggesting the complete exchange of the initial FITC-serum hard corona with the secondary population of proteins (arrows, Figure 7, row 2). Other clusters exhibit a mix of signal, suggesting that this process was still ongoing at 24 h. 

**Figure 8:**
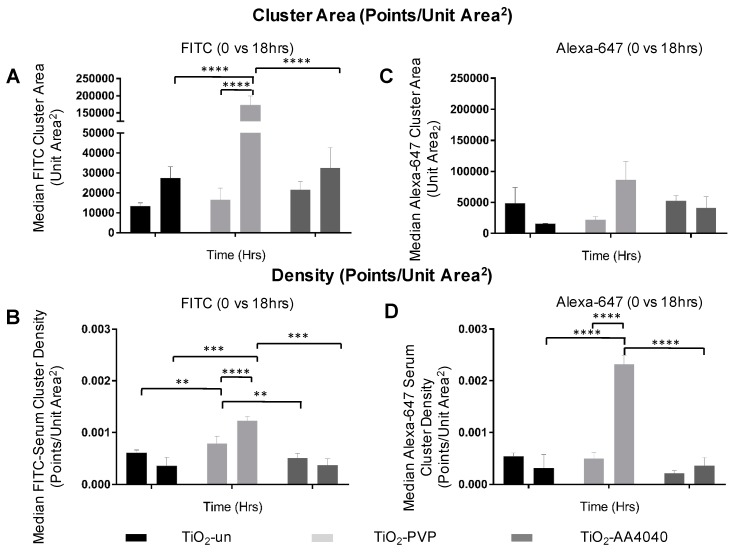
Cluster analysis of STORM data shows (**A**) a significant difference in the FITC-serum cluster size between TiO_2_-PVP and its uncoated and AA4040-coated counterparts (**** *p* < 0.0001). (**B**) Similarly, the FITC cluster density is increased at 18 h when compared to TiO_2_-un and TiO_2_-AA4040, consistent with the retention of a densely-packed FITC-serum corona. Interestingly, no significant difference in cluster area is observed in any of the observed samples (**C**); however, an increase in density is observed in the secondary Alexa-647-labeled protein at 18 hours in PVP-coated TiO_2_ (*p* < 0.0001) (**D**), suggesting the adsorption of Alexa-647-labeled protein to internalized PVP at this time point (two-way ANOVA with multiple comparisons, SEM, *n* = 3).

TiO_2_-PVP NMs demonstrated perhaps the most remarkable difference when the 0 h dSTORM image (Figure 7B) was compared to previous data. dSTORM imaging shows the presence of an abundance of small FITC-corona-associated spots which had remained undetected through the diffraction limited imaging (Biostation and confocal) previously applied in this work. Compared to other TiO_2_ NMs tested, TiO_2_-PVP maintains a high density and clusters of both FITC and Alexa-647 molecules at 18 h, suggesting that a tightly-bound protein corona is maintained throughout the observed period (Figure 7 and Figure 8), with an exchange of FITC-labeled corona with Alexa-647-labeled proteins over the observed time course. dSTORM reveals that the large agglomerates of TiO_2_-AA4040 observed in confocal imaging are actually clusters of smaller agglomerates (Figure 7).

The quantification of these data sets, comprised of millions of molecules each, was performed using the DBScan clustering algorithm (described in Materials and Methods). A statistically significant difference in cluster size and density can be observed when comparing the FITC and Alexa-647 signal to controls, indicative of a poor clustering and diffuse signal in the controls (Supplementary Figure S11). Figure 8 shows that the median cluster density and area are significantly higher at 18 h than at 0 h for both FITC (initial corona) and Alexa-647 (exchanged corona), once again supporting the formation of discrete coronas strongly associated to NMs. The data regarding the effects of surface modification on the corona formation, uptake, and degradation of TiO_2_ NMs are summarized in Table 1. Table 1 includes the quantitative details of the following findings: 1. Surface coating promotes the retention of acquired protein corona; 2. lysosomal trafficking is delayed in coated NMs, relative to the uncoated NMs; 3. the size of the intracellular NM agglomerates depends upon specific surface characteristics; 4. all conditions tested revealed an uptake of NMs by cells; 5. the degradation of intracellular NM agglomerates depends upon the specific surface characteristics.

**Figure 9:**
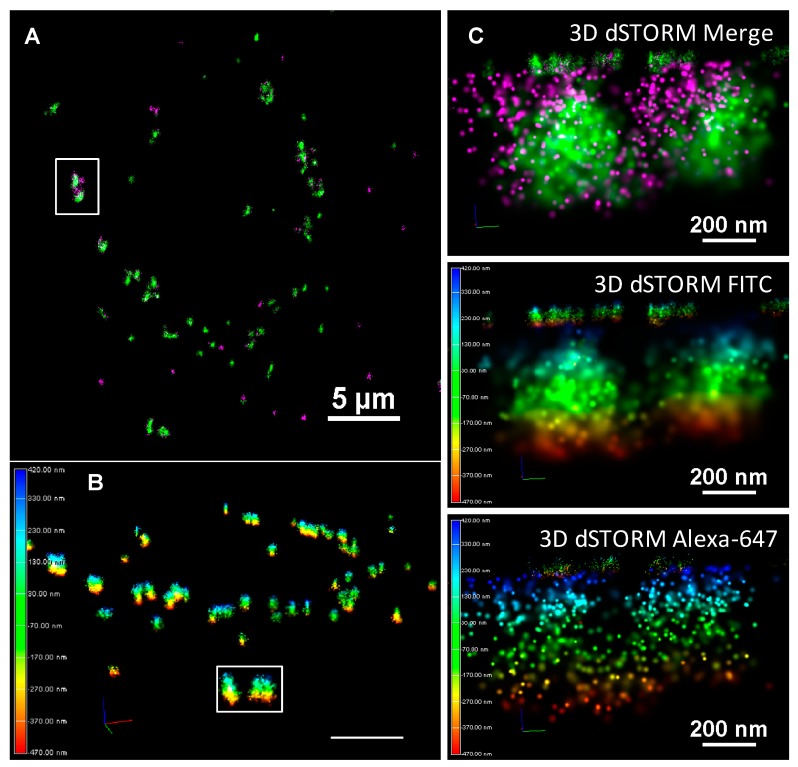
(**A**) 3D STORM imaging of the protein corona in AA4040-coated TiO_2_ NMs after 18 h incubation in A549 Cells. A 3D STORM acquisition of cells treated with TiO_2_–AA4040 shows the 3D structure of the protein corona. The green signal indicates molecules of the FITC-labeled serum constituting the initial corona, while the magenta signal is representative of Alexa-647-labeled protein comprising secondary adsorption events. (**B**) The color-coded panel represents the distribution of detections in Z, with a range of approximately 800 nm. In this manner, 3D dSTORM allows for true resolution in three dimensions. These images show discrete clusters which lie within 200 nm of one another—as such, diffraction-limited approaches will fail to resolve these as distinct objects. (**C**) In the higher zoom crop of this sample, two smaller agglomerates are observed in close proximity, with a diameter of approximately 600 nm. Of these, the initial corona appears to be comprised of tightly bound, highly localized molecules with a diameter of 300–350 nm, with the exchanged (or soft) corona (Alexa-647) forming a more loosely associated secondary layer. Image presented as a merge (with FITC and Alexa-647 in green and magenta respectively), single channel Z-color coded FITC and Alexa-647 labelled serum.

Finally, 3D STORM acquisitions of TiO_2_-AA4040-treated A549 cells at 18 h allowed for a depiction of the spatial arrangement of initial and exchanged corona proteins around TiO_2_-AA4040 NM clusters. Figure 9 is a representative image showcasing the 3D capacity of dSTORM imaging. In this example, the arrangement of a secondary layer of Alexa-647 protein around a highly localized FITC serum core can be clearly seen. Interestingly, large NM agglomerates can similarly be resolved as smaller clusters in close proximity to one another using this nanoscopic approach (Figure 8 and Figure 9).

While this dSTORM data provides unique insights into the spatial arrangement and behaviors of differently-labeled corona proteins, an important caveat to the quantification of these images lies in the distinctly different behavior of the imaged fluorophores. Different fluorophores have distinct stochastic blinking and photon counts, which impact detection and fitting. While this does not impact the final observations made in this work, it is an important consideration for future applications of this method.

It is clear that each imaging modality presented advantages and disadvantages in understanding the internalization of NMs and the evolution of their acquired protein coronas. While live cell, high throughput data from the Biostation CT allowed for the study of cells exposed to these NMs over time, the images acquired proved difficult to quantify due to the extravasation of FITC serum from cells over the incubation period. This instrument is more likely to prove beneficial in longer term studies, where cells are observed live over a period of several days. This would be particularly interesting for NMs like TiO_2_-AA4040 and TiO_2_-F127, where the initial FITC-labeled corona is retained at 24 h, in order to understand how long it persists. Despite these limitations, findings from the Biostation CT provided a basis for the further imaging and dissection of the trends observed. 

The reflectance imaging and optical sectioning capability of the confocal microscope allowed for a robust analysis of FITC-serum hard corona proteins directly associated with the reflectance signal from NMs. Reflectance confocal microscopy allowed for the accurate detection of unlabeled NMs, and therefore, the observation of these materials without the need for a directly conjugated fluorophore. For this reason, reflectance imaging can be coupled with a range of fluorescent reporters, in this instance, FITC-serum as an indicator of the initial corona and Lysotracker Red as a marker of the lysosomal compartment, for co-localization assessment.

Finally, dSTORM imaging provided a novel insight into the structure and spatial distribution of both the initial and evolved coronas, resulting from the exposure of NMs to differently-labeled populations of serum proteins. The nanoscopic resolution offered by dSTORM allowed for the resolution of small, discrete PVP-coated NM signal spots which had remained undetected in the other imaging modalities employed. Similarly, the signal that had appeared to resemble large intracellular agglomerates in the confocal images could be resolved as smaller, more discrete structures which appear to be loosely associated. This imaging modality in particular holds promise for a new level of understanding of the NM protein corona and its evolution in situ in cells. While the images presented here are a preliminary proof of principle, dSTORM has the potential for the unique quantification of the protein corona on a single molecule scale, with molecular density and clustering statistics providing an insight into corona formation and evolution kinetics on the nanoscopic scale. 

This work employed well-established models for NM–cell interactions, titanium dioxide (TiO_2_), and adenocarcinoma alveolar human basal epithelial (A549) cells, focusing on the role of surface modification and protein corona dynamics in NM cellular uptake and trafficking through a variety of microscopy modalities. Our previous work in this area demonstrated that correlative fluorescence and reflectance confocal microscopy, live cell imaging, and super-resolution microscopy methods are effective tools for studying different aspects of cellular nanomedical and cellular nanotoxicological questions [21,43,47,48]. However, this is the first study to combine microscopy modalities ranging from high-content live-cell imaging to super-resolution microscopy to address critical open questions regarding the bio-nano interface [49]. Although single-molecule localization microscopy of NMs has been performed previously by others [50], this technique has not been applied to questions concerning the roles of surface characteristics, the protein corona, agglomeration, and cellular trafficking. Our current results provide significant insights into these fundamental questions and the role of surface coating in regulating corona formation and retention. Furthermore, our super-resolution imaging provides a significantly enhanced understanding of apparent agglomerates visualized by diffraction-limited conventional microscopy methods. Finally, we have uncovered, for the first time, evidence of corona exchange inside cellular lysosomes, which contrasts with previous work suggesting that progressive corona loss through enzymatic degradation was the dominant behavior occurring following internalization and intracellular trafficking [9,25].

## 4. Conclusions

Multi-modal microscopic imaging revealed markedly different behaviors in TiO_2_ NMs with different surface chemistries in terms of their bioavailability, uptake, and cellular trafficking, as well as in the evolution of their corona proteins following internalization. Super-resolution microscopy provided a novel nanoscopic resolution of the protein corona evolution in NM-treated cells following uptake and internalization.

In the absence of surface modification, hydrophobic TiO_2_ NMs formed large extracellular agglomerates which were internalized as smaller agglomerates and efficiently trafficked to lysosomes in the 24-hour period observed. Interestingly, bare (uncoated) TiO_2_ NMs rapidly dissociated from the FITC-serum proteins used to track the initial corona following internalization, a behavior which was not seen for the various-coated TiO_2_ NMs. PVP coating results in well-dispersed TiO_2_ NMs and allows for their rapid trafficking as significantly smaller agglomerates. Of the particles observed in this study, only TiO_2_-PVP demonstrates probable degradation between 18 and 24 h (observed as a decrease in the FITC-serum intensity, reflectance area, and average particle size). Interestingly, Pluronic-F127 and Dispex-AA4040 coatings have a notable effect on the retention of the initial corona, and indeed demonstrate a partial exchange of corona proteins with intracellular proteins, as evidenced by dSTORM data.

Here, multi-modal imaging methods have been applied to provide a detailed and complex insight into how the surface chemistries used to disperse or stabilize industrially-relevant NMs directly impact their agglomeration, cellular internalization, and retention and evolution of their protein coronas. Such approaches can be conveniently partnered with toxicological and in vivo data to provide a robust basis for screening ‘safe-design’ NMs in the future. 

## Figures and Tables

**Table 1:** Summary of the observed effects of surface modification on the corona formation, uptake, and degradation of TiO_2_ NMs. Surface modification results in the retention of a hard corona, which affects the nature of particle uptake and distribution over time. Most notably, these are observed as changes in the size of internalized agglomeration and the rate at which particles are degraded (observed as a reduction in the reflectance signal after 18 h in uncoated and PVP-modified NMs).

	TiO_2_-un	TiO_2_-F127	TiO_2_-PVP	TiO_2_-AA4040
**Retention of FITC- serum corona**	No	Yes	Yes	Yes
**Lysosomal Co-localisation**	6 h (40% ± 10.3)	24 h (42% ± 12.4)	18 h (58.3% ± 8.2)	18 h (60.1% ± 26.4)
**Average internalized agglomerate size**	765.5 ± 258.2 nm	1494.0 ± 282.2 nm	354 ± 66.48 nm	6676 ± 2819 nm
**Decrease in Reflectance area (degradation)**	Yes – decrease at 18 h	No	Yes – decrease at 18 h	No

## Supplementary Materials

The following are available online at https://www.mdpi.com/2079-4991/10/3/401/s1: Figure S1: Surface Chemistries of coatings used to stabilise the TiO_2_ NMs, Figure S2: NanoMILE Standard Operating Protocol for NM dispersion in cell culture medium, Figure S3: TEM Images of TiO_2_ NM, Figure S4: DLS and ICP-MS Characterisation of TiO_2_ NMs, Figure S5: FITC-Serum binding to TiO_2_ NM versus Controls, Figure S6: Segmentation and Measurement of Biostation Images, Figure S7: Segmentation and Measurement of Confocal Images, Figure S8: Cumulative Confocal Data, Figure S9: STORM imaging Controls, Figure S10: STORM imaging increases resolution and allows for improved detection, Figure S11: Analysis of Cluster Area and Density.
