# Supplementary material for: Surface Chemistry-Dependent Evolution of the Nanomaterial Corona on TiO2 Nanomaterials Following Uptake and Sub-Cellular Localization
Source: Nanomaterials (Basel). 2020 Feb 25;10(3):401. doi: 10.3390/nano10030401 (PMC7152854; doi:10.3390/nano10030401)
Supplement: Supplementary file 1 [file nanomaterials-10-00401-s001.pdf]

Supplementary Material
for

Surface Chemistry Dependent Evolution of the Nanomaterial Corona on TiO_2 Nanomaterials Following Uptake and Sub-Cellular Localization

Abdullah O. Khan,[1] Alessandro Di Maio,[2] Emily J. Guggenheim,[3] Andrew J. Chetwynd,[3] Dan Cross,[1] Selina Tang,[4] Marie-France A. Belinga-Desaunay,[3] Steven G. Thomas,[1] Joshua Z. Rappoport,[5] Iseult Lynch3[*]

[1] Institute of Cardiovascular Science, College of Medical Dental Sciences, University of Birmingham, B15 2TT Birmingham, United Kingdom
[2] School of Biosciences, University of Birmingham, B15 2TT Birmingham, United Kingdom
[3] School of Geography, Earth and Environmental Sciences, University of Birmingham, B15 2TT Birmingham, United Kingdom
[4] Promethean Particles Ltd., 1-3 Genesis Park, Midland Way, Nottingham, NG7 3EF, UK
[5] Center for Advanced Microscopy and the Nikon Imaging Center, Northwestern University Feinberg School of Medicine, 303 E. Chicago Avenue, Chicago, IL 60611, USA
Corresponding: i.lynch@bham.ac.uk

Supplementary Figure 1: Surface Chemistries of coatings used to stabilise the TiO_2 NMs

	PVP	Pluronic® F127	Dispex® AA 4040
Chemical Structure	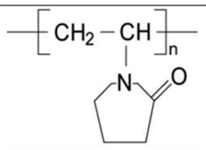	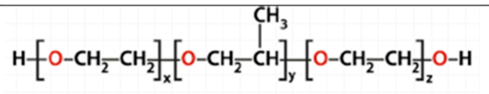	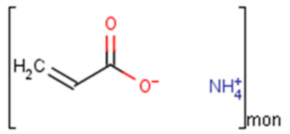
Description	Polyvinylpyrrolidone<br><br>$(C_6H_9NO)_x$	A triblock copolymer of polyethylene glycol and polypropylene glycol, $[PEG]_x$-$[PPG]_y$-$[PEG]_z$	ammonium salt of an acrylic polymer (structure shown is simplest – may have additional functionalization on the 1st carbon.
MW used	10	Average molecular weight is 12.6 kDa.	X
Charge	neutral	neutral	positive
pH		6-7	~ 7.5
pH stability range	5.0 - 8 at 10 g/L at 20 °C		5 – 10.5

Supplementary Figure : Structures of the initial set of coatings selected for surface modification of the TiO_2 NMs. PVP where n is the number of repeating units, in this case 100 as the total MW was 10,000. (b) Pluronic F127, where x, y, and z are the numbers of monomer repeats in the various blocks, in this case x and z were 99 and y is 65. (c) Dispex AA 4040 monomer unit where 'mon' indicates the monomer unit. This is an approximate structure as there may be additional substitution as BASF have not disclosed the structure Additional details are provided where available, including charge, pH of a solution and the pH stability range for thee coating.

	WP4<br>Standard Operation Procedure<br><br>Preparation of MNM dispersions for exposure of cells	Version: 01.3<br>Date: 24.03.2014<br>Page: 1 of 1

1. Equipment and reagents
1.1. Reagents
- Type 1 (ultrapure) water, sterile and endotoxin-free (e.g. autoclaved MilliQ water)
- 96% ethanol for pre-wetting of hydrophobic nanomaterials
- NanoMile centralized fetal bovine serum (FBS, Gibco, Catalogue number: 10270-106, LOT number: 41G1931K), not heat-inactivated
- Medium, pre-warmed to 37°C (the medium which is usually used for the appropriate test cell line) supplemented with 10% of NanoMile centralized serum
- chromogenic Limulus Amebocyte Lysate (LAL) test from (Lonza, QCL-1000, catalogue nr. 50-647U). Manual available under http://www.lonza.com/productsservices/pharma-biotech/endotoxin-detection/endotoxin-detection-assays/endpointchromogenic-lal-assay.aspx.

1.2. Equipment
- Microbalance with accuracy of 0.1 mg or better
- Laminar flow Biosafety cabinet to ensure sterile handling of the materials
- Sterile 15 ml conical polystyrene tubes
- Micropipettes + sterile tips
- Vortex mixer
- Ultrasonic bath (min. 250 Watt), JRC will use a vial tweeter
- For endotoxin test: sterile microvials and refrigerated microcentrifuge

2. Dispersion and dilution of powdered MNMs
- The stock dispersions and dilutions should be prepared freshly (30 – 60 min before adding to the cells). This is especially important for partly soluble MNMs like ZnO or Ag.
- weigh the powder and prepare a dispersion of 5 mg/ml in sterile water,
- vortex and ultrasonicate the dispersion for 15 min in an ultrasonic bath
- Prepare pre-warmed medium containing 10% of the NanoMile centralized serum and dilute the stock suspension (serial dilution) to the concentrations to be tested in this medium (125, 62.5, 31.3, 15.6, 7.8, 3.9, 2.0, 1.0 µg/ml). The suspension has to be vortexed vigorously immediately before taking out an aliquot.
- Mix the diluted dispersions again immediately before adding to the cells, either by vortexing or pipetting up and down.

Modification for hydrophobic material according the NanoGenotox protocol:
- After weighing the powder pre-wet the material with ethanol (96% or higher).
Calculate the ethanol volume for a 0.5% (v/v) ethanol concentration in the 5 mg/mL stock suspension.
- Proceed with the suspension procedure as described above.

1.3. Dilution of suspended MNMs (stocks > 5 mg/ml in water)
- Freshly prepare a working dispersion of 5 mg/mL in sterile water from the dispersion delivered, which has to be vortexed vigorously immediately before taking out an aliquot.
- Particle dispersions are vortexed and ultrasonicated for 5 min in an ultrasonic bath.
- Prepare pre-warmed medium containing 10% of the NanoMILE centralized serum and dilute the stock suspension (serial dilution) to the concentrations to be tested in this medium (125, 62.5, 31.3, 15.6, 7.8, 3.9, 2.0, 1.0 µg/ml). The suspensions have to be vortexed vigorously immediately before taking out an aliquot.
- Mix the diluted dispersions again immediately before adding to the cells, either by vortexing or pipetting up and down.
- If the stocks are lower concentrated than 5 mg/ml dilution can be done in medium plus 10% serum directly.

4. Endotoxin test (protocol used at KIT)
- An aliquot of the particle stock suspension (5 mg/ml in water) is centrifuged at 20,000x g for 10 min. Particles of low density e.g. polystyrene must be centrifuged for 60 min minimum.
- The supernatant is used for the endotoxin test according the instructions from the test kit supplier.

Supplementary Figure 2 NanoMILE Standard Operating Protocol for NM dispersion in cell culture medium.

Supplementary Figure 3: TEM Images of TiO_2 Particles Nanoparticles

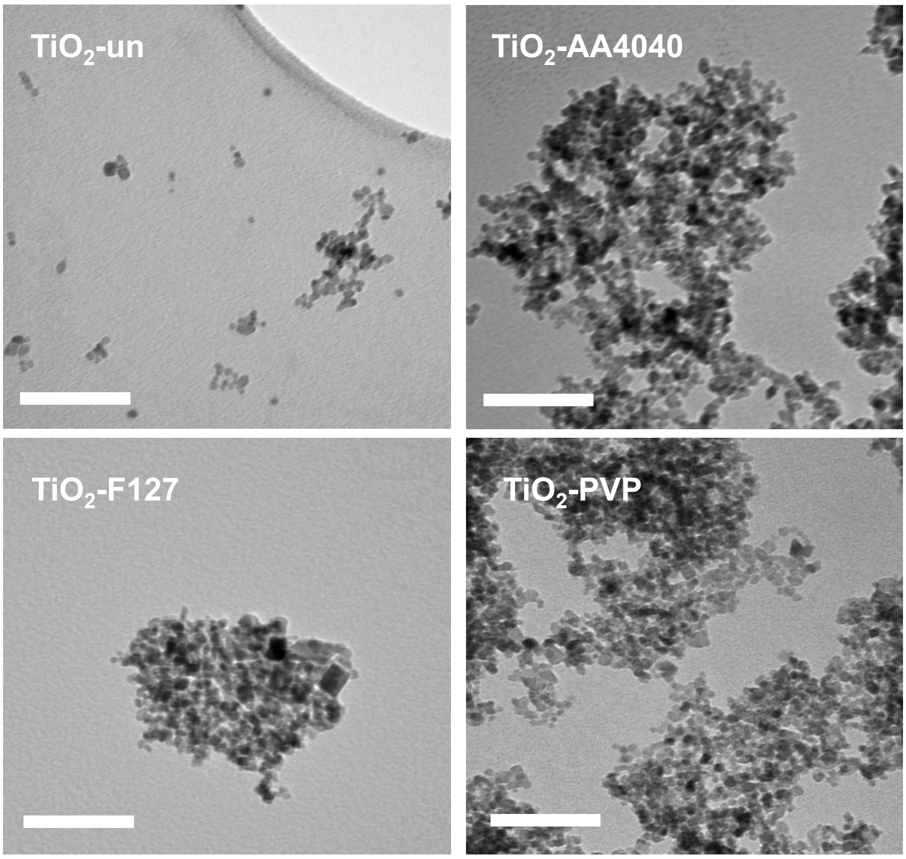

Supplementary Figure 3 TEM Images of TiO_2 NMs with different surface coatings, but similar core sizes. TiO_2-un 12 nm (± 5 nm; n=171), TiO_2-PVP 12 nm (± 8 nm; n=115), TiO_2-F127 13 nm (± 5 nm; n=116), TiO_2 AA4040 14 nm (± 9 nm; n=106).

Supplementary Figure 4:DLS and ICP-MS Characterisation of TiO_2 NPs

A

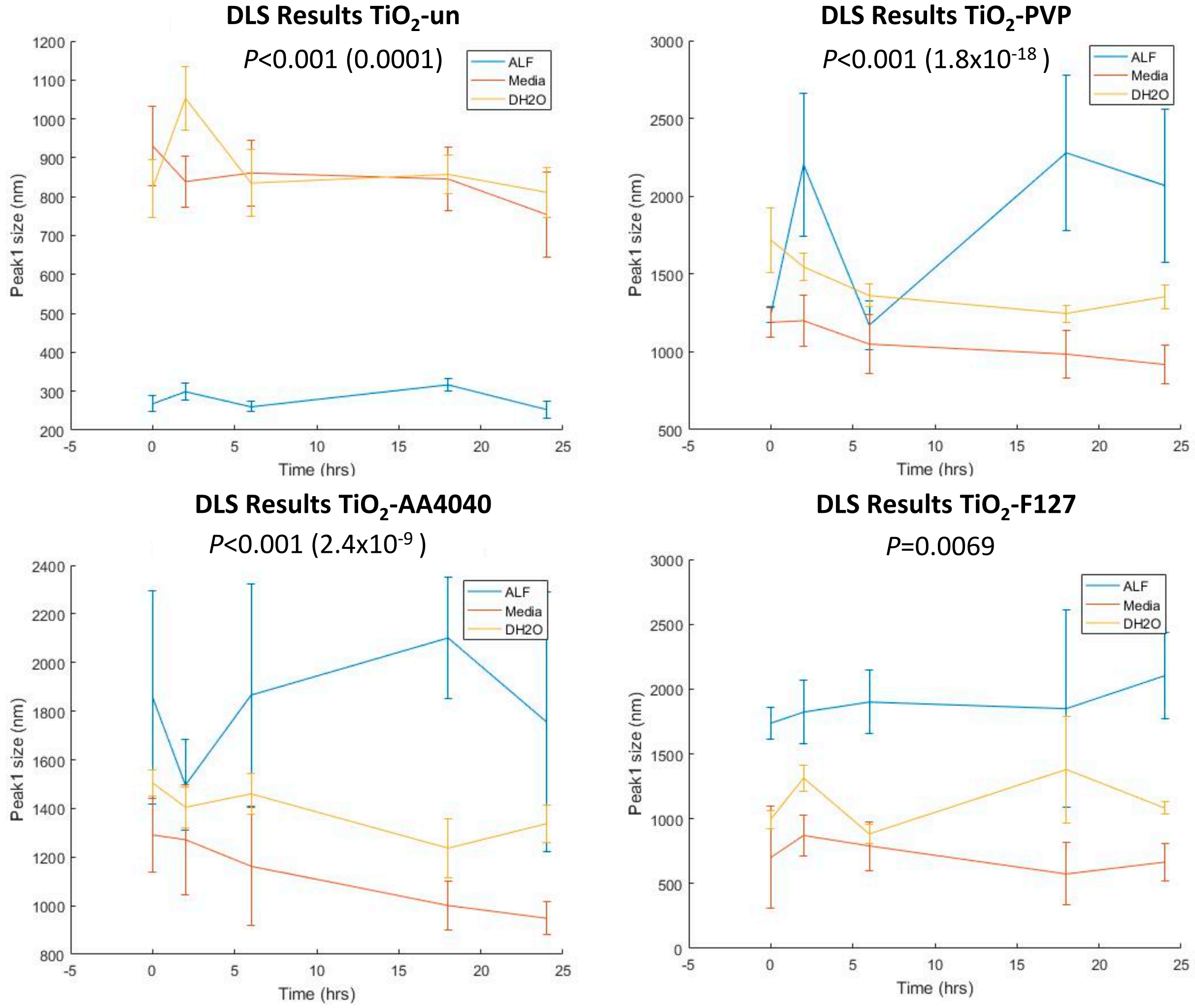

B

	Water (PPB)					ALF					SCM				
	0	2	6	18	24	0	2	6	18	24	0	2	6	18	24
Uncoated	<LOQ	<LOQ	<LOQ	0.93	0.68	57.74	82.30	130.96	208.88	393.73	<LOD	<LOD	<LOD	<LOD	<LOD
PVP	<LOD	<LOD	<LOD	<LOD	<LOD	<LOQ	<LOQ	55.03	74.62	86.63	<LOD	<LOD	<LOD	<LOD	<LOD
Dyspex	1.46	2.97	<LOD	10.40	3.98	<LOQ	<LOQ	68.73	83.8	84.07	<LOD	<LOD	<LOD	<LOD	<LOD
Pluronic	0.86	0.88	<LOQ	<LOQ	<LOQ	<LOQ	51.17	60.29	73.10	80.40	<LOD	<LOD	<LOD	<LOD	<LOD

R^2 Value for all calibrations = >0.9999. LOQ defined as 5x LOD.

Water: LOD = 0.123; LOQ = 0.615   ALF: LOD = 9.5; LOQ = 47.5   SCM: LOD = 23.717; LOQ = 115.35

C

Nanomaterial	Zeta potential (mV)
Uncoated	20.5 ± 0.497
PVP	17.3 ± 0.572
Dispex	5.3 ± 0.21
Pluronic	17.1 ± 0.497

Supplementary Figure 4: (A) Comparison of DLS measurements over time in uncoated and coated $TiO_{2.}$ Materials were incubated in media (SCM), deionised water (DH_2O), and artificial lysosomal fluid (ALF). Surface modification has a marked effect on particle size in artificial lysosomal fluid. While uncoated TiO2 demonstrates a substantially reduced size in ALF, coated particles demonstrate an increase in size when compared to incubations in complete medium and deionised water. (B) The results of the dissolution study using ICP-MS confirms the DLS data with only the uncoated TiO_2 demonstrating a large degree of dissolution which can be ascribed to the decreased size seen in the DLS. TiO2 does not show significant changes over time in the media observed, suggesting that the changes in material size and distribution are the result of cellular trafficking and active degradation. (2-Way Anova performed). (C) Comparison of zeta potentials for each nanomaterial in the study, measured in deionised water, thus likely to change upon exposure to ALF and SCM.

Supplementary Figure 5: FITC-Serum binding to Titanium Dioxide Nanoparticles vs Controls

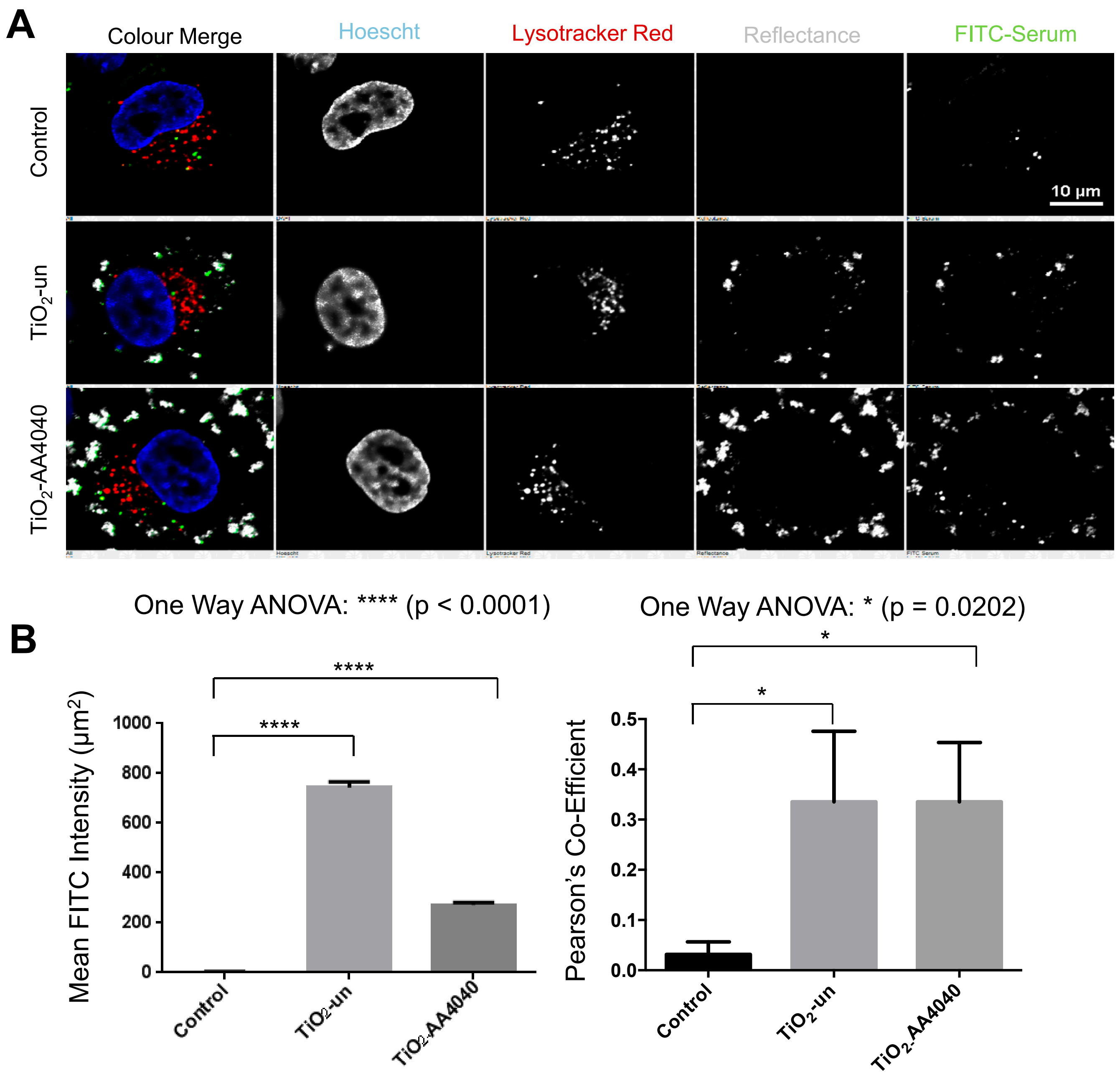

Supplementary Figure 5 (A) FITC labelling of serum containing media results in the effective labelling of the protein corona in cells treated with uncoated and dispex-44040 coated TiO_2 NMs. FITC-serum signal is significantly higher in cells treated with NMs compared to untreated controls, however some discernable non-NM associated FITC signal is detectable. **(B)** As such quantification was performed by specifically measuring FITC associated with reflectance signal, thereby robustly limiting FITC-serum measurements to the NM corona. FITC-serum signal measured was significantly different compared to controls according to a One Way ANOVA ($p < 0.0001$) n = 3. **(C)** Co-localization between FITC and Reflectance Signal was shown to be significantly higher when compared to controls (* $p = 0.0202$).

Supplementary Figure 6: Segmentation and Measurement of Biostation Images

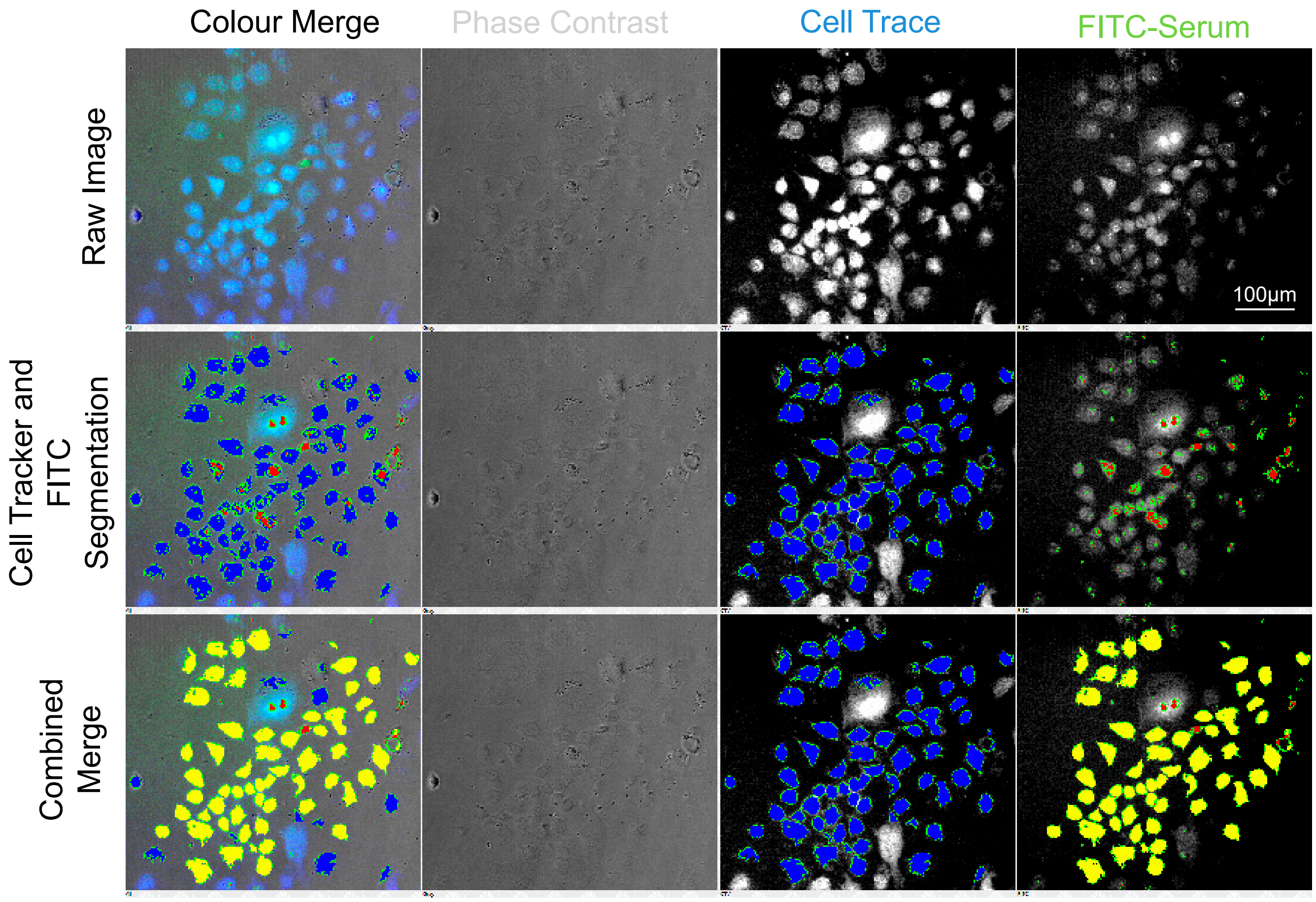

Supplementary Figure 6 Binary Segmentation and Measurement of FITC Signal within Cell Trace defined regions of interest. A549 cells treated with FITC-labelled serum are incubated with TiO_2 NMs and incubated for 15 minutes, after which cells were washed and incubated in unlabelled serum contained media and imaged over the course of 18 hours. For an effective automated analysis, cell trace and FITC signal were first de-noised and segmented to generate binaries (cell trace binaries in blue, FITC binaries in red with green outlines). Cell trace binaries were also subject to post-processing, where cells touching image borders were removed, as well as cells which could not be effectively watershed. Following this initial segmentation step, a second set of binaries were generated which were comprised of cell trace binaries positive for FITC binaries, indicated in yellow. The sum FITC intensity in these fields was calculated and subsequently divided by the total cell trace area (blue binaries). This allows for an effective, automated segmentation of the cell volume, as well as the detection of cell associated FITC signal.

Supplementary Figure 7: Segmentation and Measurement of Confocal Images

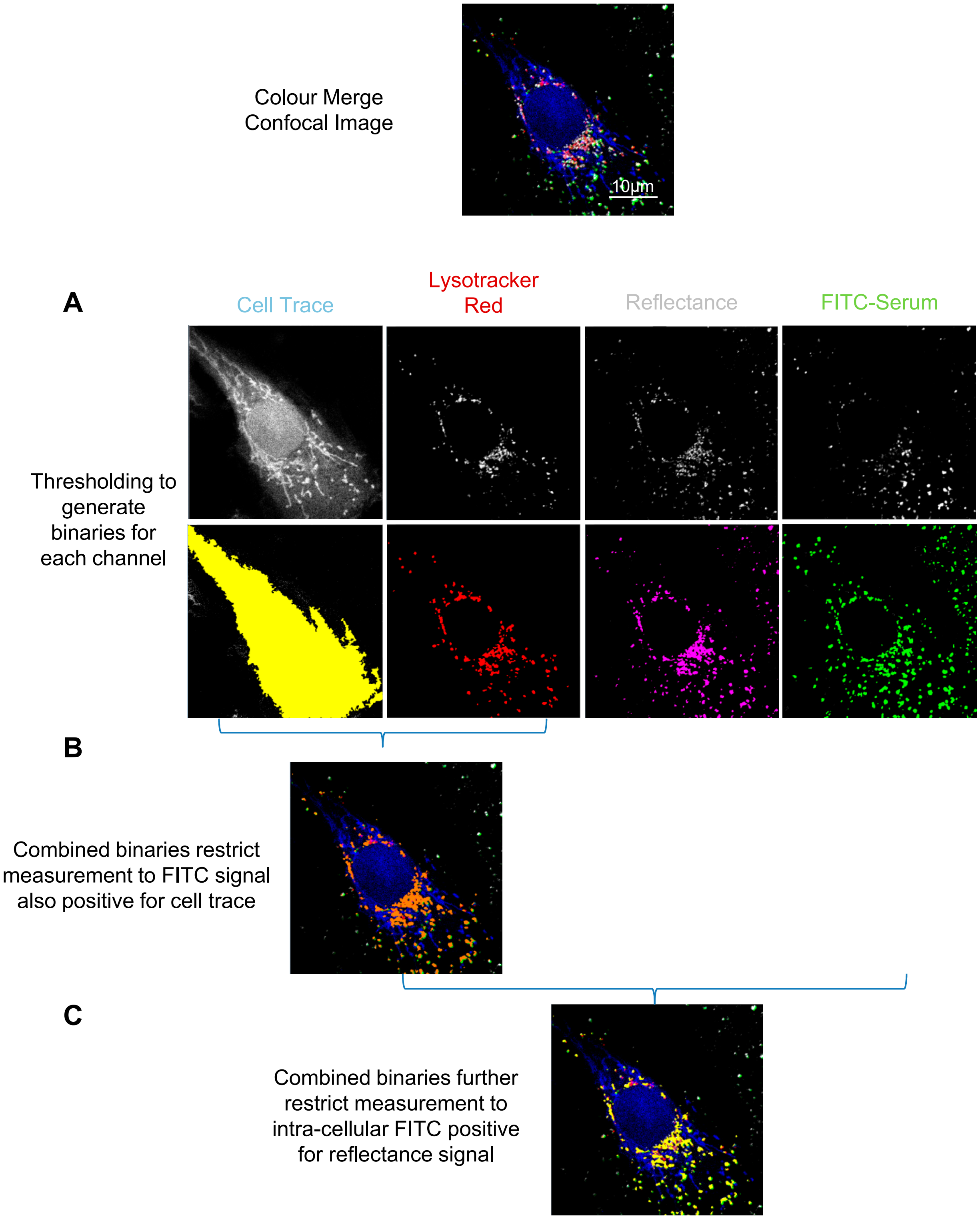

Supplementary Figure 7 Binary Segmentation and Measurement of FITC signal associated with reflectance signal in confocal fluorescence/reflectance images. Confocal images are processed through the NIS Elements High Content module by means of the General Analysis tool. **(A)** First, individual binaries are created for each channel by thresholding each plane in a Z-series. **(B)** Secondly, combined binaries are used to restrict analysis to masks within specific regions of interest. For instance, once reflectance masks have been generated for the whole field, a second binary is produced based on reflectance masks positive for Cell Trace signal, thereby allowing for a robust analysis of NMs specifically within the cell volume. **(C)** This approach can be then applied to study FITC signal specifically associated with reflectance binaries, and thereby effectively remove background signal which is not associated with NMs. By measuring these restricted binaries and normalising the subsequent intensity data to the Cell Trace measured area of the cell, comparable FITC NM corona intensities can be measured. This approach is also applied to determine the co-localisation of NMs with lysosomes, where binaries positive for both lysosomal and reflectance signal are calculated as a percentage of the total number of NMs internalised.

Supplementary Figure 8
Cumulative Confocal Data

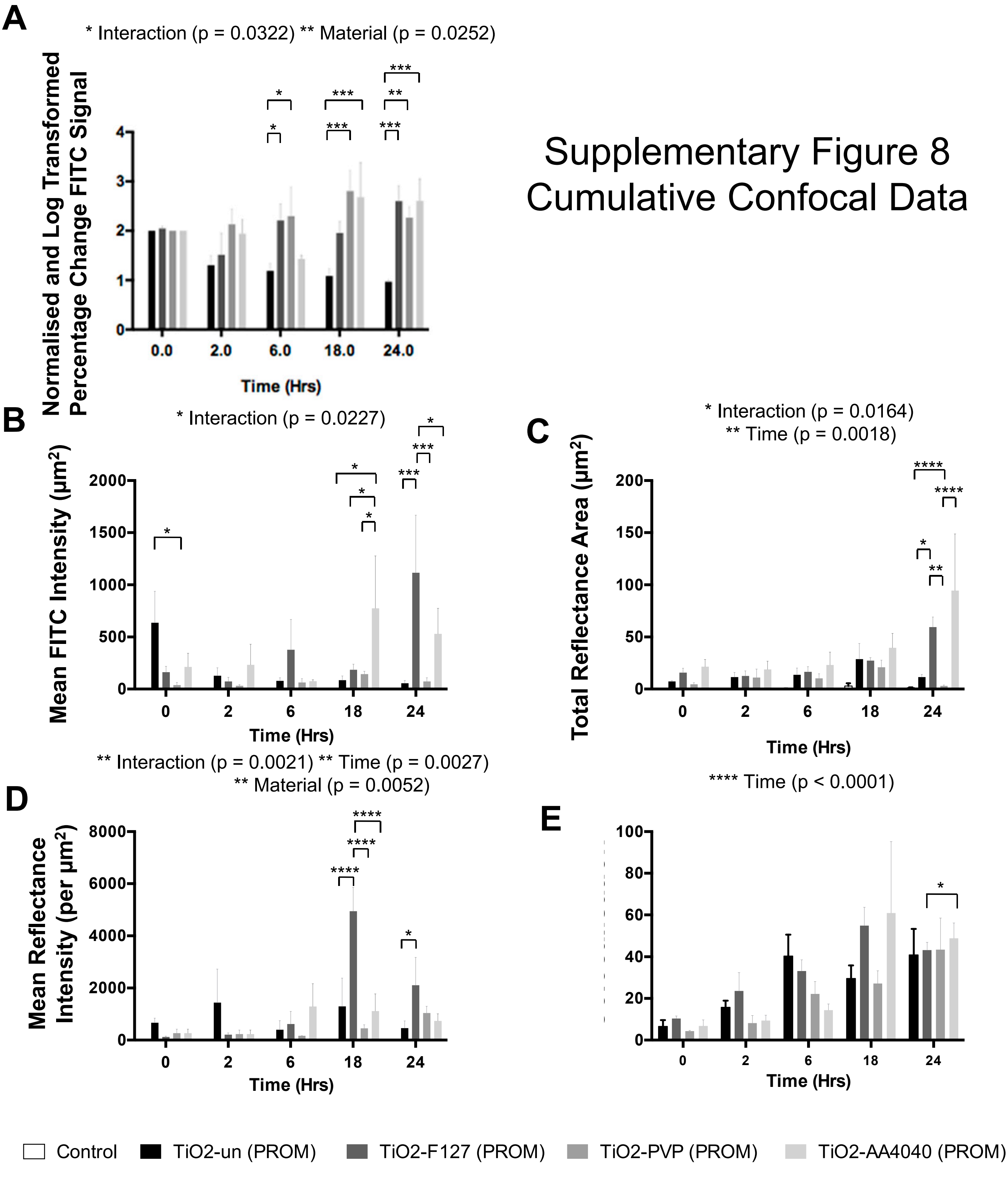

Supplementary Figure 8: Summary of confocal data. **(A)** Surface coating composition has a significant effect on the subsequent retention of FITC-Corona over time for both normalised, and raw mean FITC intensities **(B)**. **(C)** Total reflectance area observed does not vary significantly between NMIs until the 24 hour time point, at which point TiO_2-AA4040 and TiO_2-F127 both demonstrate a significant retention of NMs as large agglomerates. **(D)** Similarly, mean reflectance intensity is most significantly increased for TiO_2-F127 and TiO_2-AA4040 at 18 hours, and decreases slightly at 24, suggesting more tightly bound agglomerates at 18 hours. **(E)** Finally, no significant difference is observed in lysosomal co-localisation across TiO_2 NMs, however a significant increase is observed over time which is consistent with trafficking to these compartments.

Supplementary Figure 9: STORM imaging Controls

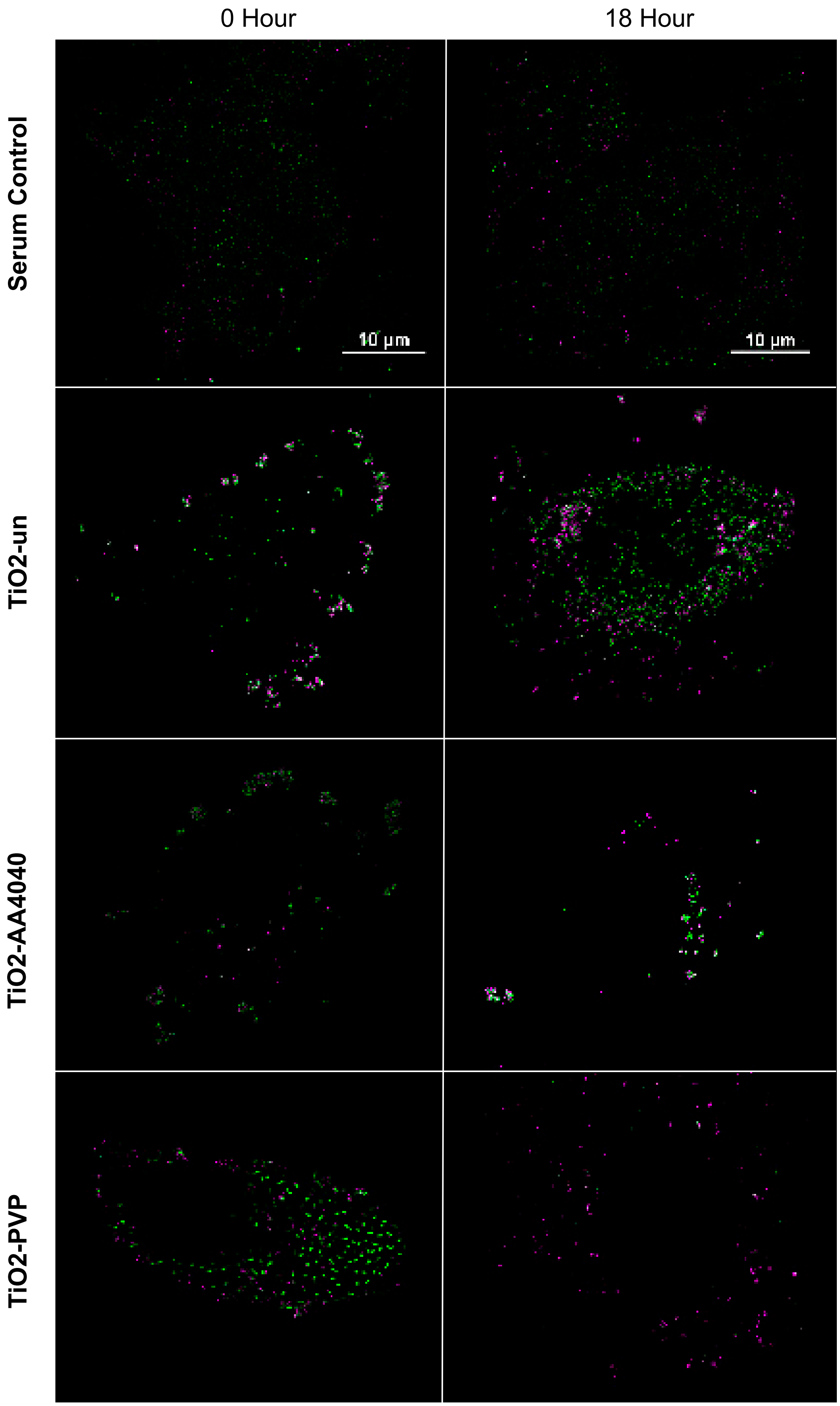

Supplementary Figure 9 STORM Imaging Controls. FITC-labelled and Alexa 647-labelled serum treated A549 cells in the absence of NMs are markedly different to TiO_2 NM treated samples. Where uncoated, PVP, and AA4040 Dispex coated TiO_2 NM treated cells demonstrate highly localised clustering comparable to confocal reflectance images in this study, control cells show a generalised and unspecific localisation within the cell. Images treated with identical density filters (25 molecules within 100nm radius).

Supplementary Figure 10: STORM imaging increases resolution and allows for improved detection

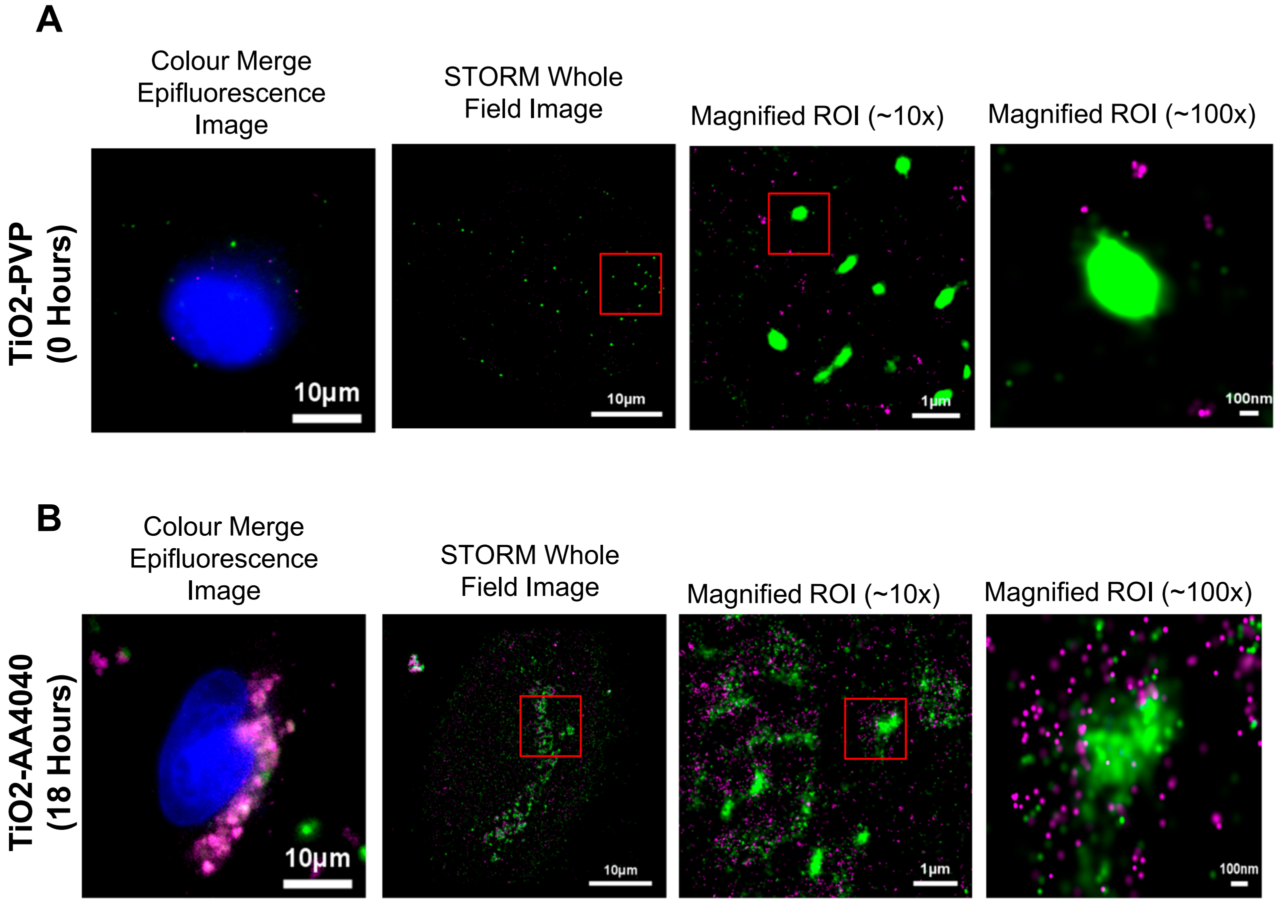

Supplementary Figure 10 (A) A549 cells treated with TiO_2-PVP NMs demonstrate previously undetectable signal compared to both confocal and Biostation CT images. **(B)** Cells treated with TiO_2-AA4040 NMs show the large aggregates previously reported (confocal and Biostation CT) at this time point (18 hours post incubation). However the increased resolution offered by dSTORM imaging allows for the resolution of aggregates on the nano-scale, showing that what were previously observed as large single aggregates can be further resolved as separate, loosely associated NM agglomerates.

Supplementary Figure 11: Analysis of Cluster Area and Density

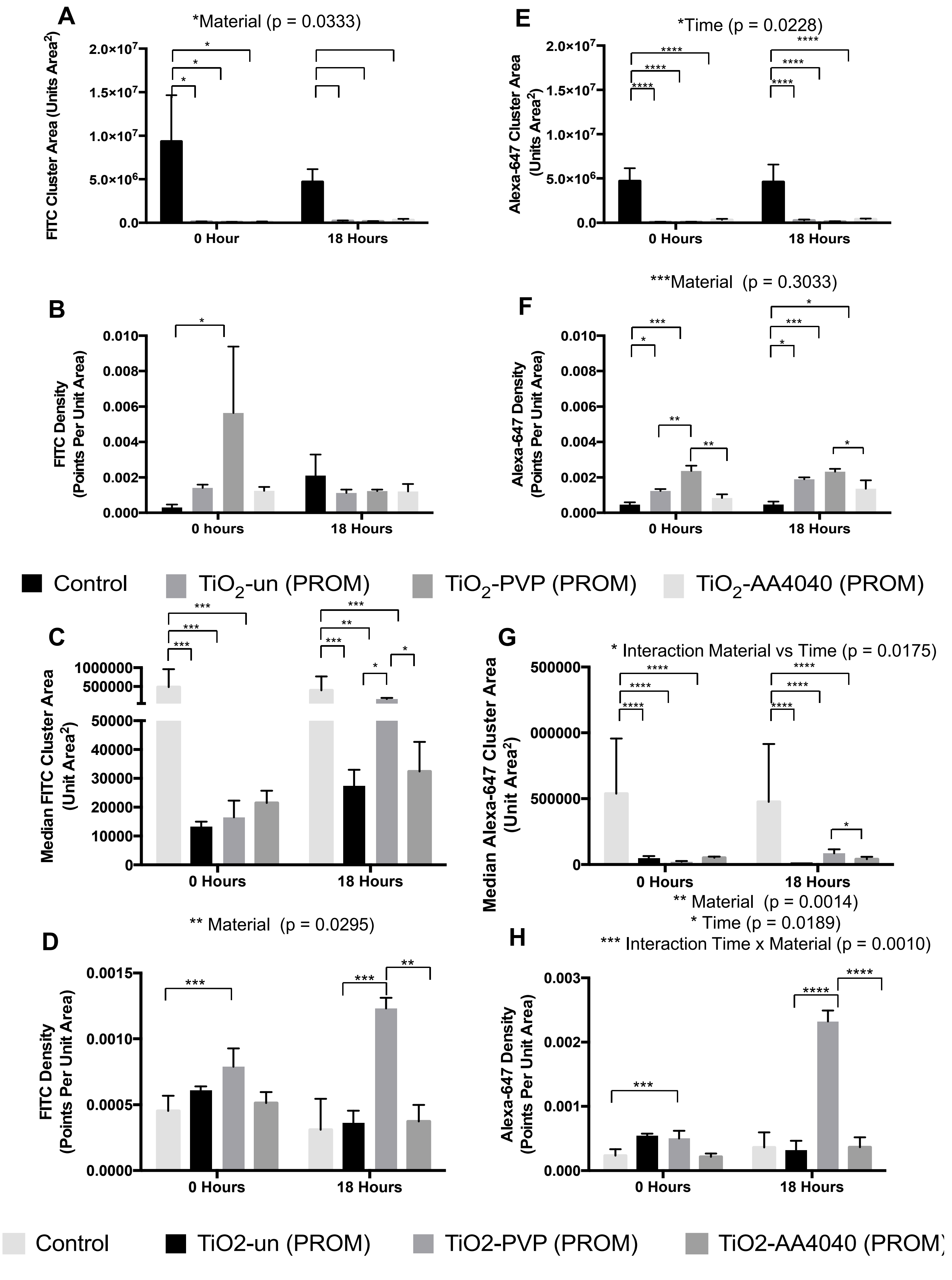

Supplementary Figure 11 (A) Cluster analysis of STORM data using the DBScan algorithm shows significant differences in FITC cluster size between controls and NM treated samples. The large cluster size is indicative of poor clustering, consistent with the diffuse signal observed in the images. **(B)** A significantly lower density in points per unit area is also observed. These findings are re-iterated where median values are plotted **(C, D)**, indicating poor clustering for control samples. Similarly, there is a significantly higher cluster area for Alexa-647 clustering **(E)**, which in conjunction with low density observed **(F)**, shows poor clustering as expected from the diffuse sample. Once more, median data supports poor Alexa-647 clustering in controls. *N = 3.*
